# Vitamin D interacts with Esr1 and Igf1 to regulate molecular pathways relevant to Alzheimer’s disease

**DOI:** 10.1186/s13024-016-0087-2

**Published:** 2016-03-01

**Authors:** Véréna Landel, Pascal Millet, Kévin Baranger, Béatrice Loriod, François Féron

**Affiliations:** Aix Marseille Université, CNRS, NICN UMR 7259, Marseille, 13916 France; APHP, Groupe hospitalier universitaire Saint Louis-Lariboisière-Fernand Widal, Centre Mémoire de Ressources et de Recherche, Hôpital Fernand Widal, Paris, France; Aix Marseille Université, INSERM UMR 1090, TAGC, Marseille, France, Marseille, 13288 France

**Keywords:** Vitamin D, Alzheimer’s disease, Transcriptome, Nervous system, Inflammatory response, Hormonal activity

## Abstract

**Background:**

Increasing evidence suggests a potential therapeutic benefit of vitamin D supplementation against Alzheimer’s disease (AD). Although studies have shown improvements in cognitive performance and decreases in markers of the pathology after chronic treatment, the mechanisms by which vitamin D acts on brain cells are multiple and remain to be thoroughly studied. We analyzed the molecular changes observed after 5 months of vitamin D3 supplementation in the brains of transgenic 5xFAD (Tg) mice, a recognized mouse model of AD, and their wild type (Wt) littermates. We first performed a kinematic behavioural examination at 4, 6 and 8 months of age (M4, M6 and M8) followed by a histologic assessment of AD markers. We then performed a comparative transcriptomic analysis of mRNA regulation in the neocortex and hippocampus of 9 months old (M9) female mice.

**Results:**

Transcriptomic analysis of the hippocampus and neocortex of both Wt and Tg mice at M9, following 5 months of vitamin D3 treatment, reveals a large panel of dysregulated pathways related to i) immune and inflammatory response, ii) neurotransmitter activity, iii) endothelial and vascular processes and iv) hormonal alterations. The differentially expressed genes are not all direct targets of the vitamin D-VDR pathway and it appears that vitamin D action engages in the crosstalk with estrogen and insulin signaling. The misexpression of the large number of genes observed in this study translates into improved learning and memory performance and a decrease in amyloid plaques and astrogliosis in Tg animals.

**Conclusions:**

This study underlies the multiplicity of action of this potent neurosteroid in an aging and AD-like brain. The classical and non-classical actions of vitamin D3 can act in an additive and possibly synergistic manner to induce neuroprotective activities in a context-specific way.

**Electronic supplementary material:**

The online version of this article (doi:10.1186/s13024-016-0087-2) contains supplementary material, which is available to authorized users.

## Background

The world is currently facing a global Alzheimer’s disease (AD) pandemic and there is an urgent necessity to find drugs that can halt, or even better, cure this pathology. Clinicians and researchers are actively looking for molecules with neuro-protective, regenerative and immuno-modulatory properties [1]. Preferably, to speed up their delivery to AD patients, the selected compounds should be FDA-approved with a well-known toxicity range.

With the intention of unveiling new molecular candidates, we elected a recognized animal model for this disease, namely the 5XFAD transgenic mice [2], that exhibit mnesic deficits, increased Amyloid Precursor Protein (APP) processing and an early onset for inflammation and plaque formation [3]. We timely assessed gene misexpression in the hippocampus and neocortex of 5XFAD female mice at presymptomatic, prodromal-like and symptomatic stages of the pathology. As expected, we observed a dysregulated expression of genes involved in inflammation, NADPH oxidase complex, phagocytic processes and interferon-γ-related pathways [4]. Remarkably, we also found a modulated expression of 784 vitamin D-associated transcripts (Additional file 1).

Vitamin D3 is a fat-soluble and seco-steroid hormone. Unlike other vitamins, vitamin D3 is mainly produced after adequate exposure to sunlight. Where this exposure is lacking, dietary intake of vitamin D (vitamin D2 or vitamin D3) is required. The epidermis contains a cholesterol metabolite that under ultraviolet light produces a precursor, previtamin D3. After isomerization and two separate hydroxylations, the active 1,25-dihydroxyvitamin D3 (1,25(OH)2D3 or calcitriol) is produced. 1,25(OH)2D3 operates *via* both nuclear receptors named Vitamin D Receptor (VDR) (part of the superfamily of steroid hormone receptors) and non-genomic systems. The 1,25(OH)2D3/VDR complex interacts with specific genomic sequences named Vitamin D Responsive Elements (VDRE) found in promoter regions and has been shown to regulate the transcription of a large number (up to 1000) of target genes [5, 6].

Epidemiological and clinical data have shown that i) high serum levels of 25-hydroxyvitamin D (25(OH)D) associate with better cognitive test performance [7, 8] and ii) vitamin D deficiency is found in patients with Alzheimer’s disease [9–13]. Other studies reported i) associations between VDR polymorphisms and cognitive function in Alzheimer’s disease patients [14–19] and ii) decreased VDR mRNA levels in the hippocampus of AD patients [20].

1,25(OH)2D3 has also been shown to i) enhance cerebral clearance of human Amyloid Beta (Aβ) peptide from mouse brain across the blood–brain barrier [21], ii) prevent Aβ-induced alterations in cortical neurons through upregulation of the VDR and downregulation of L-type voltage sensitive calcium channels [22, 23]. Experimental observations confirmed that i) vitamin D deficiency increases spatial learning deficits in a rat model of AD [24] likely by enhancing Aβ deposition through modulation of amyloid processing [25] and ii) vitamin D3 supplementation decreases pathological markers of the disease, such as Aβ deposition, in a transgenic mouse model [26]. However, relatively little is known about the mechanisms of action of this steroid hormone in demented brains.

Purposely, we assessed the therapeutic benefit of vitamin D3 in 5XFAD mice, supplemented after the onset of the symptoms, from month 4 (M4) to month 9 (M9). Along the course of the disease, we measured memory abilities and, at the end of the experiment, we quantified amyloid plaque load. In addition, using pangenomic cDNA microarrays and bioinformatic tools, we identified the genes that were regulated, directly or indirectly, by a vitamin D3 intervention. This study is the first to provide insight into the molecular mechanisms at play after chronic treatment with vitamin D3 in both healthy aging and AD-like brains.

## Results

### Vitamin D3 supplementation induces an extensive gene dysregulation

**Fig. 1:**
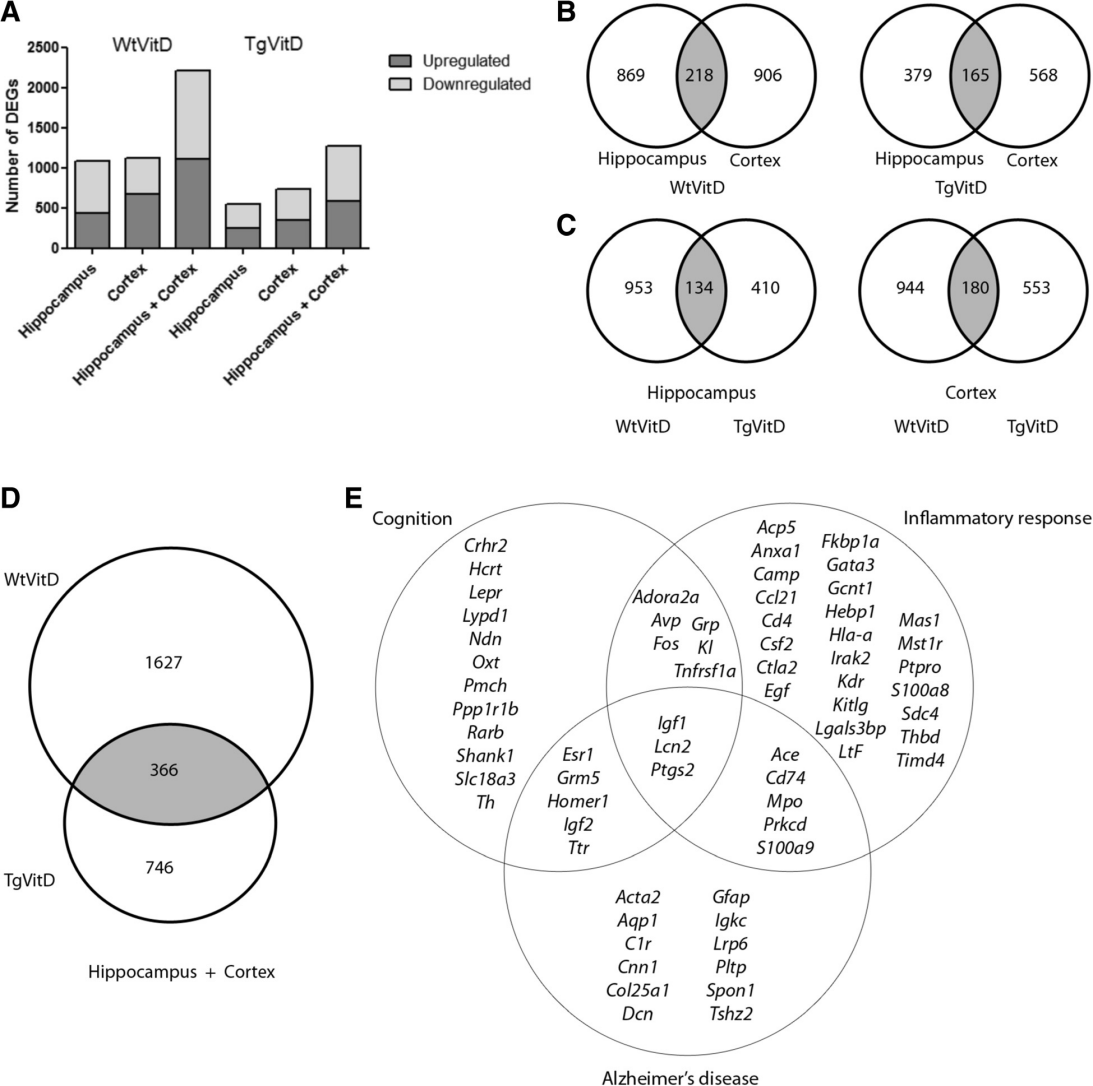
Schematic overview of gene expression in cortex and hippocampus of 9 month-old wild type and transgenic mice reveals that a 5 month vitamin D3 supplementation induces an extensive transcript dysregulation. **a** Graphical representation of the number of upregulated (*dark grey*) and downregulated (*light grey*) DEGs in either the hippocampus, cortex or combined regions in both Wt and Tg animals having been fed a vitamin D3 diet for 5 months. The number of genes specifically dysregulated in wild type mice is twice as large as in transgenic mice. **b**-**c** Venn diagrams indicating the number of overlapping (*grey*) and non overlapping DEGs when the two brain areas are compared in wild type and transgenic mice (**b**) and when the two strains are compared at the hippocampal or cortex level (**c**). **d** Venn diagram showing that, when hippocampal and cortical data are combined, vitamin D3 supplementation triggers the dysregulation of 366 genes in both strains. **e** List and top associated biological functions of the 366 DEGs common to both strains. Overall, 39 are associated to inflammatory response, 26 to cognition and 25 to Alzheimer’s disease. Fold change (FC) cutoff used for above analyses was -1.5> FC <1.5

To reveal the potential molecular targets of vitamin D3 in specific brain regions, we conducted a series of transcriptomic experiments. The study allowed a comparison of dysregulated genes and associated molecular pathways affected by vitamin D3 supplementation in both a non-AD and AD context, *i.e*. Wild-type (Wt) versus Transgenic (Tg) animals. Figure 1 reveals the total number of transcripts affected in Wt or Tg animals after 5 months of vitamin D3 supplementation. A total of 2211 genes are dysregulated in Wt animals in both the hippocampus and cortex combined, compared to a total of 1277 Differentially Expressed Genes (DEGs) in Tg mice after vitamin D3 treatment (Fig. 1a). Vitamin D3 supplementation induces a dysregulation of nearly twice as many genes in Wt animals as compared to Tg. Details about these DEGs are given in Additional file 2. There is no significant difference in terms of the number of upregulated or downregulated genes in both genotypes and brain regions (Fig. 1a). When considering each brain area analyzed, there is little difference in the number of dysregulated genes for a given genotype (Fig. 1b). In Wt mice, about 10 % of DEGs are common to both tissues while 13 % shared DEGs can be observed in Tg animals (Fig. 1b). Interestingly, a number of DEGs are common to both genotypes in both brain areas (Fig. 1c). Up to 366 genes are commonly dysregulated after vitamin D3 treatment in both Wt and Tg mice (Fig. 1d). Using Ingenuity Pathway Analysis (IPA) software, the clustering of the 366 genes indicates that among the top canonical pathways, the ones displaying the largest number of DEGs are: Axon guidance signaling (13 genes), Protein kinase A signaling (13), ILK signaling (9), Huntington’s disease signaling (9), Glioblastoma Multiform signaling (8), Cdc42 signaling (8), IL8 signaling (8), Agranulocyte adhesion and diapedesis (8), Tight junction signaling (7), Embryonic stem cell pluripotency (6), Corticotropin Releasing Hormone signaling (6), Communication between innate and adaptive immune cells (5), Glutamate receptor signaling (5) and Dopamine receptor signaling (5). Furthermore, 39 out of 366 genes are associated to inflammatory response, 26 to cognition and 25 to Alzheimer’s disease (Fig. 1e).

### An intriguing expression of hormone transcripts in the cortex and hippocampus

One of the aims of this transcriptomic analysis was to extract information about genes whose expression is modified in the nervous system after 5 months of vitamin D3 supplementation, regardless of the pathological context. One way to obtain such information is to refine the working dataset to include only genes that are commonly dysregulated in all tested conditions. Table 1 shows the list of genes found to be dysregulated in Wt and Tg mice, in both the hippocampus and cortex. In these four conditions, we found 28 DEGs, with varying fold changes according to the genotype and brain region studied, that are consistently regulated after 5 months of vitamin D3 supplementation. To gain better sense of the biological functions represented by the differentially expressed genes, we performed a Functional Annotation Clustering using the NIH David tool. This analysis revealed that the genes in this dataset are related to five different biological clusters: regulation of phosphorylation, hormone activity, membrane trafficking, nucleotide binding and metal ion binding. A less restrictive additional analysis was performed on DEGs found in at least three out of the four conditions. Table 2 represents the top ten biological clusters associated with this dataset. One hundred thirty-six genes were differentially expressed (Additional file 3) and when performing functional annotation clustering of these DEGs, the number one biological function is hormone activity (Table 2). Eight genes fall into this ontological category: *Avp, Igf1, Igf2, Nppa, Oxt, Pmch, Prl, Trh.* Bearing in mind that several of these neuropeptides are largely found in the pituitary axis, it is extremely intriguing to find them differentially expressed in the cortex or hippocampus of 5XFAD mice.

**Table 1:** List of commonly dysregulated genes in the cortex and hippocampus of transgenic and wild type mice

		Fold change			
		Hipocampus		Cortex	
Gene symbol	Entrez gene name	WtVitD/Wt	TgVitD/Tg	WtVitD/Wt	TgVitD/Tg
*Ahcy*	adenosylhomocysteinase	−1,73	−2,07	1,79	2,07
*Atg4c*	autophagy related 4C, cysteine peptidase	−2,72	−2,75	4,49	4,82
*Avp*	arginine vasopressin	−1,59	2,21	1,73	3,96
*C130078n14*	uncharacterized protein C130078N14	−2,13	−2,17	3,92	2,77
*Cdk5rap1*	CDK5 regulatory subunit associated protein 1	−1,78	−2,14	2,66	2,92
*Cpne1*	copine I	−1,56	−1,82	1,6	1,71
*Dnajb8*	DnaJ (Hsp40) homolog, subfamily B, member 8	−1,85	1,98	−1,92	−1,73
*Fam205a*	family with sequence similarity 205, member A	2,34	2,78	1,61	−1,55
*Fkbp1a*	FK506 binding protein 1A, 12kDa	1,97	1,62	−1,72	−1,52
*Gbp6*	guanylate binding protein family, member 6	2,19	2,21	−2,65	−3,23
*Gm4924*	predicted gene 4924	1,68	1,94	2,14	1,53
*Hla-drb5*	major histocompatibility complex, class II, DR beta 5	1,86	1,53	−2,53	−2,34
*Iqcf4*	IQ motif containing F4	2,74	2,22	−1,54	−1,73
*Iqsec3*	IQ motif and Sec7 domain 3	−5,23	4,49	−3,3	1,58
*Kcnj6*	potassium channel, inwardly rectifying subfamily J, member 6	1,61	1,93	−1,79	−1,67
*Kdr*	kinase insert domain receptor	−1,6	1,68	1,53	−1,64
*Mirg*	miRNA containing gene	−1,91	1,88	−3,41	−1,68
*Nanog*	Nanog homeobox	−2,91	1,53	−1,52	−1,66
*Ocel1*	occludin/ELL domain containing 1	−3,07	−3,98	3,52	3,21
*Omp*	olfactory marker protein	−1,54	−10,41	3,18	1,66
*Oxt*	oxytocin/neurophysin I prepropeptide	−2,75	4,04	2,03	4,74
*Ppp1r16b*	protein phosphatase 1, regulatory subunit 16B	2,29	2,39	−1,78	−1,78
*Prkcd*	protein kinase C, delta	−2,18	−2,52	1,84	1,65
*Prl*	prolactin	1,93	−1,88	10,07	10,54
*Rnase4*	ribonuclease, RNase A family, 4	1,61	2,12	−2,14	−2,07
*Sdc4*	syndecan 4	2,38	1,95	−1,91	−1,81
*Serpina3g*	serine (or cysteine) peptidase inhibitor, clade A, member 3G	2,78	1,94	−1,8	−2,42
*Znf669*	zinc finger protein 669	−2,08	−1,9	1,84	2,2

Dysregulated transcripts in the hippocampus and cortex of vitamin D3 supplemented wild type and transgenic mice in comparison with unsupplemented littermates. The gene symbol, the full name and the fold change of each gene are indicated

**Table 2:** Top ten biological clusters associated with transcripts dysregulated in at least 3 out of 4 conditions: misexpression in the hippocampus and/or cortex of transgenic and/or wild type mice

Annotation term	Number of genes	Genes
Hormone activity	8	*Avp, Igf1, Igf2, Nppa, Oxt, Pmch, Prl, Trh*
Synaptic vesicle	5	*Doc2g, Slc17a6, Syn3, Sv2c, Syt10*
Regulation of phosphorylation	6	*Cd4, Cd74, Cdk5rap1, Fkbp1a, Irak2, Prkcd*
Membrane-bounded vesicle	8	*Capn11, Doc2g, Gpnmb, Prl, Slc17a6, Syn3, Sv2c, Syt10*
Striated muscle tissue development	4	*Fkbp1a, Foxp1, Rxrg, Tnnt2*
Blood circulation	4	*Acta2, Ace, Avp, Nppa*
Lymphocyte activation	4	*Cd4, Cd74, Fkbp1a, Foxp1*
C2 calcium-dependent membrane targeting	4	*Cpne1, Doc2g, Prkcd, Syt10*
Immune effector process	4	*Cd74, C1qb, Foxp1, Prkcd*
Synapse	5	*Shc4, Slc17a6, Syn3, Sv2c, Syt10*

Cluster name, number of genes within each cluster and acronym of each gene are indicated

Among the top ten biological pathways affected by vitamin D3 treatment, two of them concern synaptic processes and neurotransmission with dysregulation of genes such as *Syn3, Syt10* and *Sv2c*, while two others are related to immune responses including genes such as *C1qb, Cd4* and *Cd74* (Table 2). A literature search was then performed to determine whether some of these genes contain a putative VDRE in their sequence. Among the 136 genes commonly dysregulated, only four, *Fos, Gcnt1, Irak2, St6galnac1,* were found to present a known functional VDRE in their promoter region [6].

### The effects of vitamin D3 supplementation vary according to the health status of the animal

**Fig. 2:**
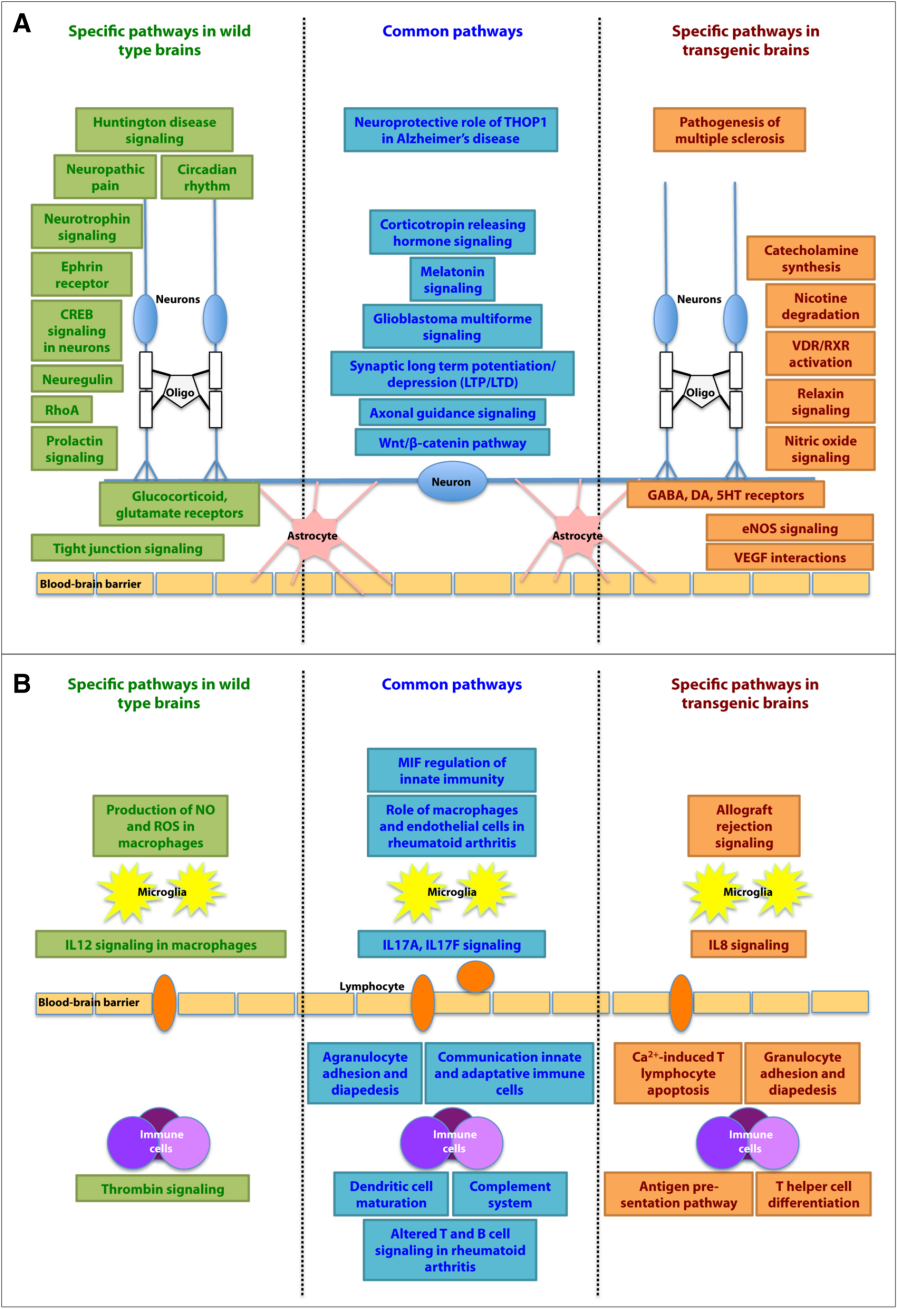
Top canonical metabolic pathways associated to gene dysregulation in the cortex and hippocampus of mice after 5 months of vitamin D3 supplementation: common versus strain-specific processes. Metabolic pathways associated to gene expression dysregulation were identified using Ingenuity Pathway Analysis (IPA). Data from both the cortex and hippocampus were analyzed as one dataset and the main metabolic pathways affected were clustered according to their association to the nervous (**a**) or the immune (**b**) system

To move further and comprehensively assess the effect of vitamin D on brain functioning, we analyzed the record of dysregulated genes, in accordance with their role in well-described metabolic pathways. For each strain, we combined transcriptomic data from the hippocampus and the neocortex at M9 and, using Ingenuity software, we listed the main canonical pathways that were modified by the vitamin D3-enriched diet. We then compared the two strains of animals (Wt *vs* Tg). All perturbed pathways and the names of their associated DEGs are recorded in Additional file 4. Out of the 90 major modified canonical pathways, 42 were common to both strains. As shown in Fig. 2, 16 of them are related to either the nervous or the immune system.

In regard to the brain (Fig. 2a), the largest number of dysregulated genes – 53 and 38 for the Wt and Tg animals, respectively – is associated to Axon guidance signaling. Within both strains, we observe a modified expression of growth factors (e.g. *Bdnf, Bmp1, Bmp6, Egf, Igf1, Figf, Ntf3, Vegfc*), chemo-attractive/repulsive agents (e.g. *Sema3b, Sema3c, Sema4g, Sema6b, Sema6c*), proteases (e.g. *Ace, Adam11, Adamts2, Adamts8, Adamts9, Mmp9*) and TGF/WNT/β-catenin-associated molecules (e.g. *Tgfb1, Tgfbr1, Tgfbr2, Wnt1, Wnt3, Wnt6, Wnt9a, Wnt9b, Wnt16*). Two other metabolic pathways - the signaling of the corticotropin releasing hormone and the multiform glioblastoma – include large subsets of dysregulated genes. However, the highest ratio (number of dysregulated genes/number of genes involved in the pathway) is found for the neuroprotective role of THOP1 in Alzheimer’s disease.

With respect to the immune system (Fig. 2b), three canonical pathways – Role of macrophages, fibroblasts and endothelial cells in rheumatoid arthritis, Agranulocyte adhesion and diapedesis and Dendritic cell maturation - emerge as primarily affected by vitamin D3 supplementation, in both strains. Other modified metabolic pathways include Communication between innate and adaptive immune cells, Complement system and MIF regulation of innate immunity. Nonetheless, the peak ratios are observed for the Role of Il17F in inflammatory diseases and the role of Il17A in psoriasis.

In addition to this strain independent response to vitamin D3 supplementation, the brains display specific modifications in line with the health status of the animal. For example, vitamin D3-enriched diet modify the expression of glucocorticoid and glutamate receptors in wild type animals and GABA, dopamine and serotonin receptors in transgenic animals (Fig. 2a). Similarly, vitamin D3 alters interleukin signaling in a strain-dependent manner. IL-12 and IL-8 pathways are adjusted in Wt and Tg brains, respectively (Fig. 2b). Other examples of discrepancies can be listed. The non pathological brain responds to vitamin D3 treatment by modifying the expression of i) nitric oxide in macrophages, ii) neurotrophins and iii) molecules related to Huntington disease whereas the pathological brain reacts by altering the expression of iv) nitric oxide in endothelial cells, v) cathecholamines and vi) molecules associated to Multiple Sclerosis.

### Vitamin D3, a potent upstream regulator altering the expression of Alzheimer’s disease-associated genes

To further decipher the detailed role of vitamin D in brain functioning, we narrowed our analysis to the genes that are directly or indirectly regulated by vitamin D or its related-metabolites. Using Ingenuity software, we listed all dysregulated molecules whose expression has been associated, in the literature, to this steroid.

**Fig. 3:**
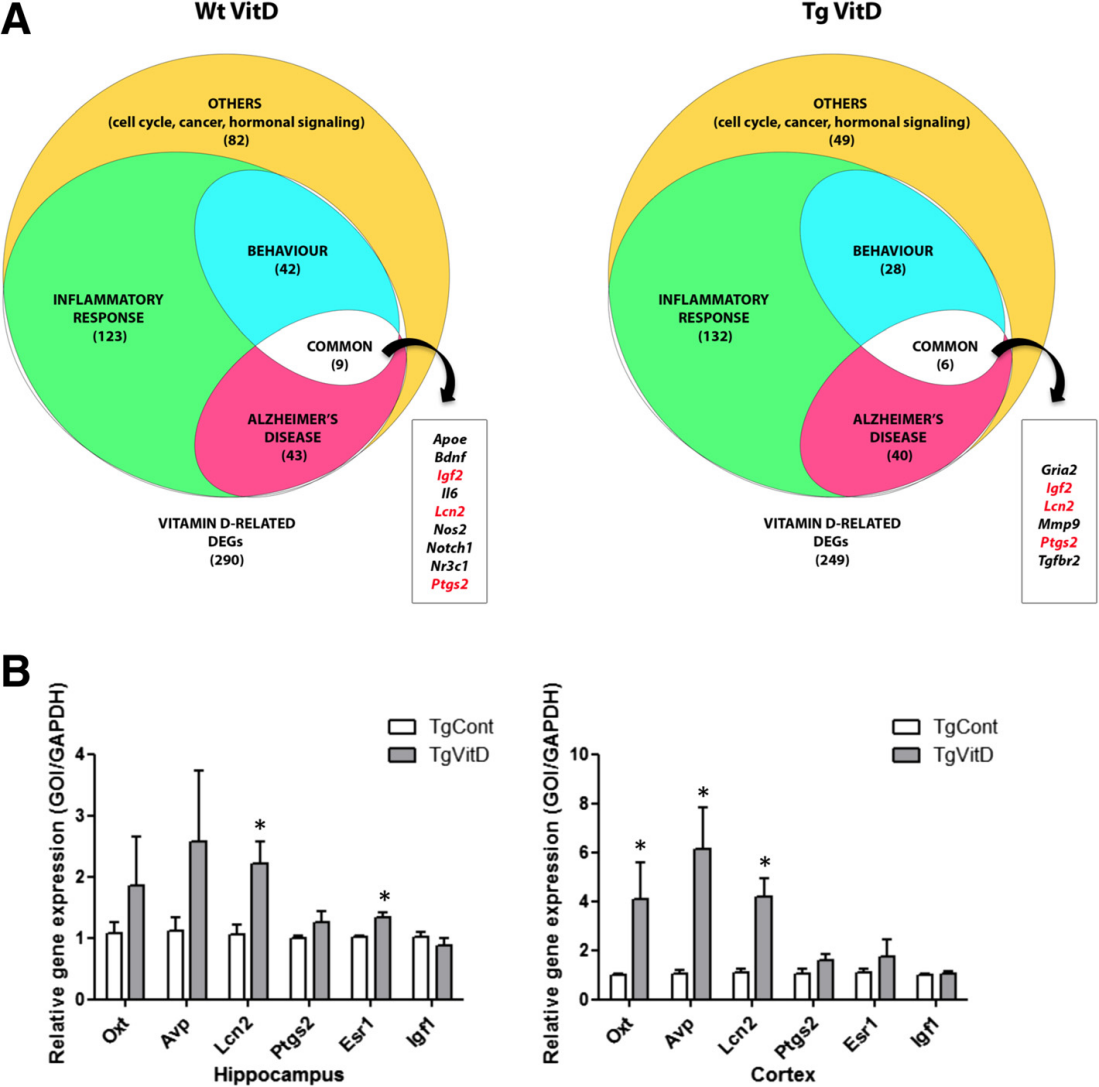
Analysis of vitamin D3-related dysregulated genes indicates an association with inflammatory response, Alzheimer’s disease and behavior. **a** Overall, 290 and 249 vitamin D-related DEGs are found misexpressed when comparing vitamin D3-supplemented wild type and transgenic mice with their unsupplemented littermates, respectively. Functional classification of these DEGs show importance of inflammatory, behavioural and Alzheimer’s disease related processes. **b** qPCR validation of several dysregulated transcripts (*n* = 6 per group). An overexpression of the transcripts oxytocin (*Oxt*) and arginine vasopressin (*Avp*) is confirmed in the cortex as well as an upregulation of estrogen receptor 1 (*Esr1*) in the hippocampus. The gene coding for lipocalin 2 (*Lcn2*) is overexpressed in both the hippocampus and the cortex. * = *p* < 0.05

As summarized in Fig. 3, we observed 290 and 249 vitamin D-related DEGs respectively, when comparing vitamin D3-supplemented Wt and Tg mice with their unsupplemented littermates. Within these two pools, 188 and 151 genes are part of the initial list of 784 vitamin D-associated transcripts whose expression is altered in the brain of transgenic animals (Additional file 5). Furthermore, when assessing the presence of a VDRE in the promoter region of these DEGs [6], we found 25 (13 %) and 18 (12 %) transcripts in the two above-mentioned populations (highlighted in grey in Additional file 5). As a comparison, 68 (9 %) out of the 784 genes listed in Additional file 1 harbor a VDRE. We then clustered the two populations of vitamin D-related DEGs and, as shown on Fig. 3, we highlighted three sub-populations, composed of transcripts involved in inflammatory response, Alzheimer’s disease and cognition.

At this point, we decided to validate the observed expression of certain DEGs of our dataset, using quantitative PCR (qPCR). We chose two hormones, *Oxt* and *Avp*, which are, according to the microarray results, dysregulated in the hippocampus and cortex of both Wt and Tg animals. We also focused on four vitamin D-related genes involved in AD commonly dysregulated in the neocortex and hippocampus of Tg animals, namely *Lcn2, Ptgs2, Esr1* and *Igf1*. An overexpression of the transcripts *Oxt* and *Avp* is confirmed in the cortex as well as an upregulation of *Esr1* in the hippocampus. The gene coding for *Lcn2* is overexpressed in both the hippocampus and the cortex. However, the downregulation of *Igf1* is not confirmed by qPCR analysis (Fig. 3b).

**Fig. 4:**
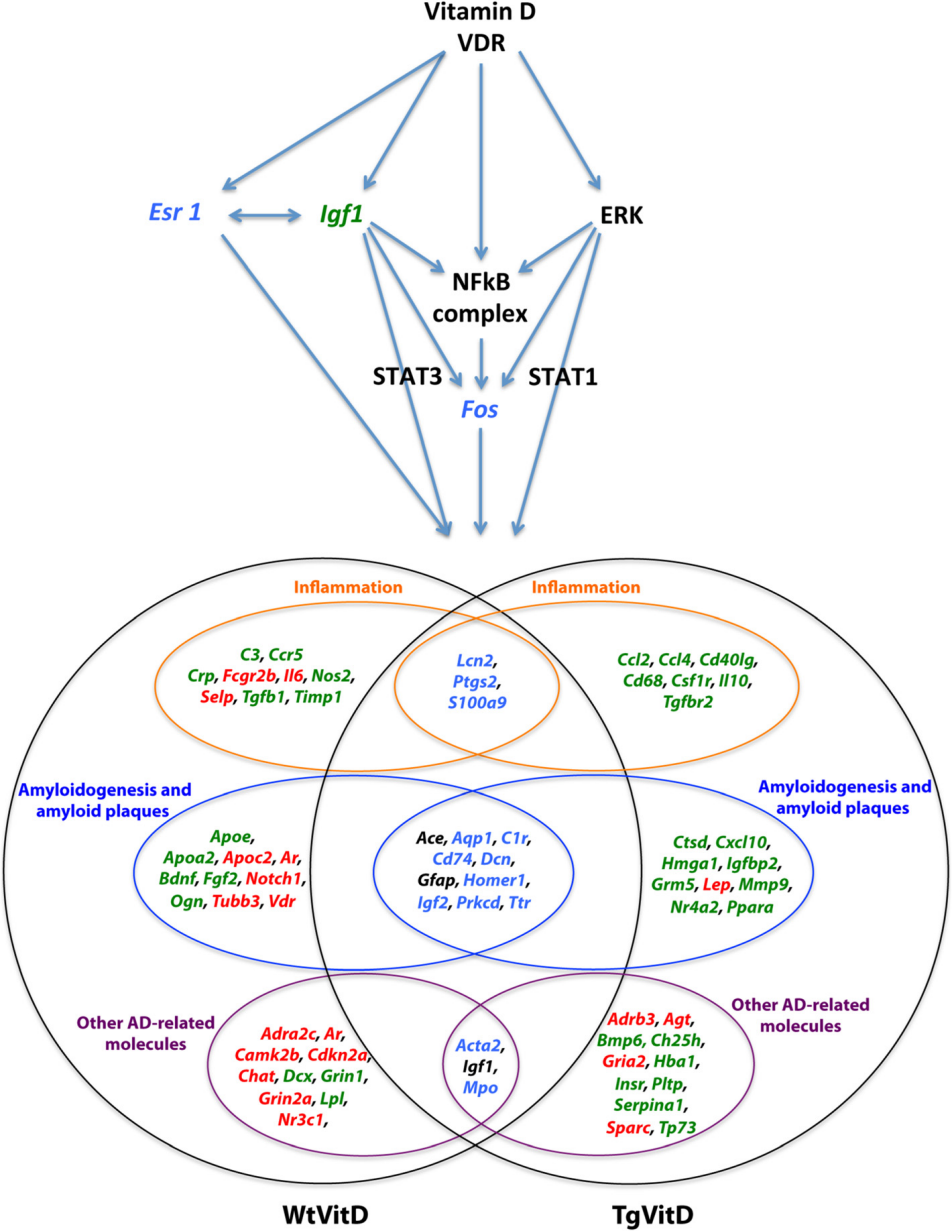
Schematic view of the putative mechanisms of vitamin D3 action in the brain. Vitamin D3 or its related metabolites and their receptors act via four main regulators: IGF1, ESR1, ERK and NF-κB. *Igf1* is underexpressed (*green*) in both strains while *Esr1* is overexpressed in transgenic brains and underexpressed in wild type brains (*blue*). The expression of *Erk* and *NF-κB* is unchanged in both strains but many transcripts under their control are misexpressed in transgenic and wild type animals. Downstream gene regulators include *Fos*, *Stat1* and *Stat3. Fos* is overexpressed in transgenic animals and underexpressed in wild type animals (*blue*). Overall, 72 vitamin D-related transcripts are associated to either inflammation (19 genes) or amyloidogenesis and amyloid plaques (29) or AD-related features (24). Sixteen genes are dysregulated in both strains (*center areas*). However, only three of them - *Ace*, *Igf1* and *Gfap* - are similarly misexpressed (*black colour*). Specific up- (*red*) and down-regulated (*green*) are indicated for each strain

Vitamin D3 supplementation alters the expression of many Alzheimer’s disease-associated genes, even in Wt brains. However, major differences can be observed. As reported on Fig. 4, 29 and 27 transcripts are uniquely misexpressed in Wt and Tg animals, respectively. In addition, 13 out of the 16 vitamin D-related DEGs emerging at the intersection of both strains are inversely expressed. The three transcripts that are similarly misexpressed are *Ace, Igf1* and *Gfap*, strongly related to Alzheimer’s disease. All three are underexpressed when compared to unsupplemented animals (Fig. 4).

The consequences of this discordant response to vitamin D3 treatment are unknown since the Wt mice display no AD symptoms and features. Nevertheless, it allowed us to envision the putative mechanism of action of vitamin D3 or its related metabolites (Fig. 4). Vitamin D3 or 1,25(OH)2D3 and its receptor likely act *via* four regulators, namely IGF1, ESR1, ERK and NF-κB. *Igf1* is underexpressed in both strains while *Esr1* is overexpressed in Tg brains and underexpressed in Wt brains. The expression of *Erk* and *Nf-κb* is unchanged in both strains but many transcripts under their control are misexpressed in Tg and Wt animals. Downstream gene regulators include *Fos, Stat1 and Stat3*. The former is overexpressed in Tg and underexpressed in Wt animals.

### Vitamin D3 treatment impacts amyloid plaque load and gliosis in transgenic animals

**Fig. 5:**
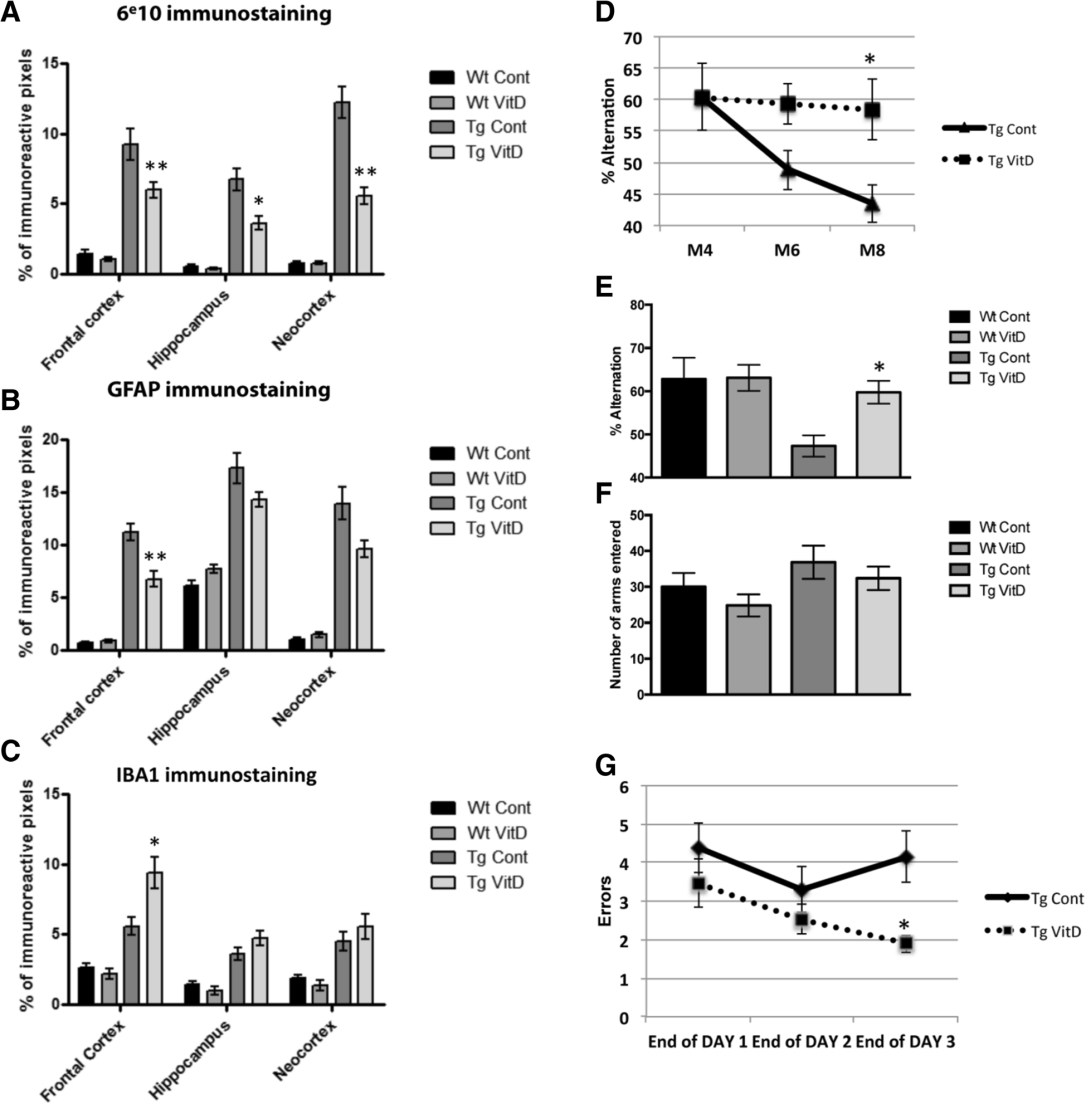
Vitamin D3 supplementation reduces amyloid plaque load and cognitive deficits in transgenic animals. **a** In the three assessed brain regions - frontal cortex, hippocampus and neocortex – the number of amyloid plaques is reduced in transgenic supplemented brains. Vitamin D3 treatment limits astrogliosis (GFAP staining) in the frontal cortex and neocortex (**b**) and increases microglial activation (IBA1 staining) in the frontal cortex (**c**) of transgenic mice. Cognitive performance was assessed using the Y-maze and the radial arm water maze. **d** Vitamin D3 supplemented transgenic mice do not display the decreased percent of alternation (Y maze) observed in unsupplemented transgenic mice over time as measured at M4, M6 and M8. **e**, **f** At the end of the supplementation period (M8), a significant decrease in percent alternation performance is observed in transgenic mice. This effect is rescued by vitamin D3 supplementation with no significant difference in the number of arms entered. **g** Using the radial arm water maze, hippocampal-dependent spatial working memory was assessed. At the end of the learning process (Day 3), a significantly reduced number of errors is observed in supplemented transgenic mice. * = *p* < 0.05; ** = *p* < 0.01

In order to examine whether a 5-month therapeutic intervention with vitamin D3 translated into a positive functional outcome, animals were analyzed at the histological and behavioral levels for known markers of AD pathogenesis. At the histological level, vitamin D3 supplementation decreased the quantity of plaques in three different brain regions of Tg mice: frontal cortex, hippocampus and neocortex (Fig. 5a). Astrogliosis, assessed through GFAP-immunostaining, is greatly increased in Tg animals compared to Wt. Vitamin D3 treatment of Tg animals during 5 months significantly decreases GFAP reactivity. However, this significant decrease is only observed in the frontal cortex (Fig. 5b). Microglial activation was assessed through immunostaining with IBA1 antibody and although there is an increase in this microglial marker between Wt and Tg control mice, vitamin D3 supplementation increases IBA1 immunoreactivity only in the frontal cortex of Tg animals when compared to their unsupplemented littermates (Fig. 5c).

### Four months of vitamin D3-enriched diet is sufficient to rescue cognitive deficits in the 5XFAD model

After 4 months of vitamin D3-supplemented diet, cognitive performance was evaluated using two different paradigms: the Y-maze and the 6-radial arm water maze. A longitudinal study was first performed from M4 to M8 using the Y-maze. This study shows that Tg animals display a decreased percent of alternation with time whereas aging Tg animals on a high vitamin D3 diet do not show any cognitive decline between M4 and M8 (Fig. 5d). At M8, we observed a significant decrease in percent alternation performance in Tg mice. This effect is rescued by treatment with vitamin D3 during 4 months with no significant difference in the number of arms entered (Fig. 5e and f). However, a high vitamin D3 diet does not improve working memory in wild-type animals, even at M8 (Fig. 5e). The RAWM was used in addition to the Y-maze to assess hippocampal-dependent spatial working memory and reference memory. At the end of the learning process (end of day 3), results show a significant decrease in the total number of errors for Tg animals having been fed a vitamin D3 supplemented diet compared to Tg animals on a control diet (Fig. 5g).

## Discussion

Despite growing evidence that vitamin D deficiency is a risk factor for cognitive decline and possibly Alzheimer’s disease, very few studies have investigated the effect of vitamin D supplementation in AD mouse models. To our knowledge, this is the first transcriptomic study to assess the effect of long-term vitamin D3 supplementation in the nervous system of an AD mouse model. This work allowed us to examine the effects of chronic vitamin D3 treatment in both normal aging and AD-like brains, at the transcriptomic, histological and behavioral levels. We observed a very large number of dysregulated genes in both the pathological and non AD-like cortex and hippocampus of these animals at M9. On the one hand, our results point out that, although there are nearly double the number of DEGs in Wt animals compared to Tg, a high number of genes misexpressed after vitamin D3 supplementation are common to both genotypes. On the other hand, when considering the biological pathways affected by these DEGs, the common pathways between Wt and Tg often include different subsets of genes for a given pathway or an inversed expression for the same gene. Our study underlines some well-known actions of vitamin D and related metabolites, namely its impact on immune and inflammatory processes and cell cycle, along with previously shown effects on nervous system molecules such as neurotrophins and nitric oxide. However, our results also comprise unexpected effects on the expression of hormonal transcripts and reveal regulation of genes involved in synaptic function, axon guidance, endothelial and vascular processes and stem cell pluripotency. A reassuring result is that among the 784 genes predicted to be related to vitamin D action in Tg animals, almost a third of them are indeed regulated after 5 months of supplementation. On top of that, the majority of the vitamin D-related genes are associated to Alzheimer’s disease, in both the Wt and Tg brains. We show that vitamin D action in the brain of this AD mouse model likely involves signaling through ESR1 and IGF1 and that a 5-month therapeutic intervention reduces the histological markers of the disease and restores learning and memory capacities.

### A common set of brain-associated genes regulated by vitamin D

As mentioned earlier, no pangenomic study previously assessed the effect of vitamin D3 supplementation in a mouse model of Alzheimer’s disease. As a result, no direct comparison can be performed. However, three teams performed a thorough transcriptomic analysis on i) brains from adult rats that were vitamin D-deprived during gestation [27] ii) 1,25(OH)2D3-supplemented cultures of neurons and glia [28] or pericytes [29] and iii) hippocampus from middle aged rats after chronic treatment with increasing doses of vitamin D3 [30]. A cross examination of our data with those reported in these studies reveals that the dysregulated expression of 46 genes – *Aaas, Aldoa, Atp2b4, Bcam, Bmp1, C3, Cat, Ccl2, Cntn4, Col16a1, Dpp6, Epas1, Fam120c, Fam70a, Fbln1, Fkbp1a, Foxc1, Gfap, Grp, Hspa12a, Htr2c, Igf1, Kdr, Kif1a, Lcat, Lpl, Ndst4, Nmbr, Nr4a2, Pde3b, Pmch, Polr1b, Ppp2r2b, Rab33b, Sdc4, Senp7, Serinc2, Slc17a6, Syt2, Thbd, Tia1, Trhr, Ttc39c, Vcam1, Vdr and Vps13c* – was formerly observed. The newly generated list may appear relatively restricted when compared to our initial dataset of 3488 dysregulated genes. However, given the variety of scientific paradigms (depletion *vs* supplementation), the nature of the samples (cells vs different brain regions), the molecular diversity of the administered molecule (vitamin D3 *vs* 1,25(OH)2D3) and the heterogeneous size of the datasets, it is not of minor importance to be able to establish an inventory of commonly misexpressed genes in nervous cells or brain areas after vitamin D3 treatment. Moreover, some studies have performed targeted analysis of specific gene expression in nervous system cells or tissues. For instance, Kaneko and collaborators demonstrated that calcitriol regulates the expression of two human brain-related genes containing VDREs, tryptophan hydroxylase (Tph) and leptin (Lep) [31]. These genes are also modulated in the brains of either Wt or Tg mice in our study. In a set of experiments examining the effect of maternal vitamin D deficiency on fetal brain development, Hawes and colleagues found that the pups from deficient mothers display a modulated expression of *Bdnf, Foxp2, Tgfb1* and *Th,* which are also affected in certain conditions of our study [32]. Altogether, these results underline the necessity for additional work to be performed on the genomic effects of vitamin D3 or 1,25(OH)2D3 in nervous system cells, as there are clearly multiple targets relevant to brain function and these could potentially explain a causal relationship between vitamin D status and brain aging or AD.

We chose to perform a literature-based analysis of possible VDRE-containing DEGs in our datasets. To avoid discrepancies between study designs, we initially based our search on a large *in silico* and microarray-based study by Wang and colleagues [6]. This revealed the presence of a very limited number of putative VDREs in our dataset. However, although ChiP-seq and genome-wide studies have found that the most frequent sequence located in VDR binding regions is the classic consensus VDRE motif, these data also suggest the presence of other motifs whose configuration diverge from the classic one and that might or might not interact directly with the VDR [33]. Moreover, there appears to be 1000–10,000 possible chromatin VDR binding sites, according to the cell-type considered. In contrast the number of primary 1,25(OH)2D3 target genes is thought to be around 100–1000 per tissue, suggesting that some genes are controlled by more than one VDR binding site [33]. It is therefore difficult to conclude on the complete list of VDR direct targets in our dataset. For instance, the study by Latimer and coworkers, reported putative VDRE sequences in three genes from our dataset – *Cntn4, Ppp2r2b and Syt2 -* which were not previously identified by Wang and colleagues [6, 30]. These potential VDRE-containing DEGs are involved in synaptic vesicle trafficking and neurotransmission, indicating a possible direct modulation of these processes by 1,25(OH)2D3 in the 5XFAD animal model. Since the determination of true VDRE sequences would require further investigation and that vitamin D metabolites are also known to interact with various membrane-based signaling pathways, the search for indirect actions, for instance through modulation of immune processes, is not to be excluded.

### A confirmed immuno-modulatory role, several mechanisms of action

The impact of vitamin D3 on inflammatory and immune processes is all but unexpected since it is now well established that vitamin D is a potent immune modulator [34]. 1,25(0H)2D3 adjusts the expression of inflammatory cytokines and prevents the proliferation of proinflammatory cells [35]. As a general rule, 1,25(OH)2D3-treated T-cells produce less proinflammatory Th1 (IL-2, interferon-γ, tumor necrosis factor-α), Th9 (IL-9) and Th22 (IL-22) cytokines and more anti-inflammatory Th2 (IL-3, IL-4, IL-5, IL-10) cytokines [36–39]. In addition, Th17 activity is affected by vitamin D. Activated T-cells treated with 1,25(OH)2D3 secrete reduced levels of IL-17, interferon-γ and IL-21 [40]. The overall picture remains slightly blurry. However, several key regulatory mechanisms begin to surface.

Characteristically, when calcitriol binds the VDR/RXR, the complex recognizes VDREs located in the promoter region of target genes and activates or inhibits their transcription. Concerning IL8 signaling, one of the top commonly affected processes in both Wt and Tg animals in our study, it is likely that IL8 is under the primary control of 1,25(OH)D and VDR as a study recently demonstrated the presence of VDR binding sites, in human monocytes, within the interleukin 8 (IL8) cluster [41]. However, direct activation/repression of gene expression is not the only mechanism by which the vitamin D system can act on immune processes [42]. As presented in Fig. 2, IL17A and IL17F signaling are affected by vitamin D3 supplementation in both strains. No known VDREs are present in the promoter regions of the TH17-signature cytokines that are *Il17a* and *Il17f*. Recently, it was reported that vitamin D inhibition of Th17 differentiation is obtained through modulation of Smads. More specifically, the presence of a negative VDRE in *Smad7* promoter leading to *Smad7* repression along with a VDR-dependent increase in *Smad3* explain the changes observed in Th17 cells during differentiation. In parallel, 1,25(OH)2D3 indirectly regulates the expression of Th17-specific genes *Il17a* and *Il17f* through activation of ERK [43]. On top of direct transcriptomic action and indirect signaling through vitamin D receptors, it is also possible that epigenetic mechanisms are also at play. For example, 1,25(OH)2D3 regulates TLR-mediated inflammation signaling by downregulating miR-155 in macrophages [44]. In our study, the modulated expression of several members of the TLR family (*Tlr1, Tlr2* and *Tlr7*), with no known VDREs, could potentially involve such mechanisms [45].

In addition to these various mechanisms, 1,25(OH)2D3 interacts with other transcription factors, such as the glucocorticoid receptor, the nuclear factor of activated T-cells (NFAT) or NF-κB resulting in overall decreased inflammatory states [46]. It has been shown that, when activated by its ligand, the VDR dimerizes with the glucocorticoid receptor and together they bind to glucocorticoid responsive elements of genes, leading to an up-regulation of anti-inflammatory molecules and a down-regulation of pro-inflammatory genes [47]. Glucocorticoid receptor signaling is one of the top biological functions affected in our study, in both Wt and Tg brains. VDR is also able to bind to an NFAT binding site, inducing a diminished NFAT activity and downstream a repressed expression of IL-2, cyclooxygenase 2 [48] and IL-17 [49]. Similarly, 1,25(OH)2D3 has been shown to inhibit NF-κB activation and signaling through up-regulation of IκB expression [50], interference with DNA binding [51] and by enhancing FBW-7 dependent turnover of NF-κB subunits [52], leading to a reduced production of pro-inflammatory cytokines and pro-inflammatory enzymes such as cyclooxygenase 2 [53]. Deciphering the exact mode of action for 1,25(OH)2D3 modulation of immune and inflammatory pathways, whether it involves direct gene regulation, interactions and cross-talks between various signaling pathways, and epigenetic mechanisms is beyond the scope of this study. However, it is clear that in both the healthy and AD-like brain, vitamin D3 supplementation induces a strong effect on inflammatory and immune processes.

### A background-specific action of vitamin D3

As established during the last 25 years, vitamin D and its metabolites play crucial roles in cell proliferation and differentiation, neuroprotection, neurotransmission and neuroplasticity [54]. However, as observed in the immune system, vitamin D3 action is space- and time-specific. For example, a hypervitaminosis D alters the synthesis of calcium binding proteins in the caudate/putamen but not in the hippocampus and the cerebral cortex [55]. Likewise, 3 weeks after the induction of an experimental allergic encephalomyelitis, vitamin D3 supplementation provokes a massive downregulation of inducible nitric oxide synthase (iNOS) only in the rat cerebellum and brainstem [56]. The time window for vitamin D depletion or supplementation can be essential as well. A transient developmental vitamin D deficiency induces persistent morphological and molecular changes in the adult rat brain [57–59] while an adult vitamin D deficiency is associated with less profound modifications [60].

An important finding from our transcriptomic analysis is the effect of the pathological context on gene response to vitamin D3 treatment. Indeed, our study provides evidence that vitamin D3 has distinct genomic effects in a non-pathologic and an AD-like brain. It is clear that, when given to healthy animals, vitamin D3 induces dysregulation of genes implicated in an important number of nervous system related pathways, whereas it appears to act preferentially on immune system processes in Tg mice, as illustrated in Fig. 2 and Additional file 4. When considering nervous system related pathways, distinct actions also appear. For instance, the non-pathologic brain expresses genes associated to neurological functions such as neurotrophins or tight junction modulation while the pathologic brain activates/represses genes related to catecholamine synthesis or nitric oxide signaling (Fig. 2). Another mean to demonstrate the effect of the pathological background on gene dysregulation is to observe the subtle strain-associated changes in a common canonical pathway. One of the most remarkable is the Wnt/β-catenin signaling. Out of seven genes whose expression is dysregulated in both strains – *Dsh, Frizzled, Lef/Tgf, Lrp1, Rar, Tgfbr, Wnt* – 5 are inversely misexpressed, indicating a putative opposite action of vitamin D3. In addition, 12 and 7 genes are exclusively dysregulated in the wild type and transgenic brains, respectively (Additional file 6).

Several studies have highlighted the importance of various factors, such as genetic settings, pathological backgrounds, organ areas, cell subtypes, delivery time windows, molecule concentrations, in determining the response to vitamin D treatment. We can list a few examples. Conversely to cells from healthy individuals, 1,25(OH)2D3 increases the production of IL-2 in peripheral blood mononuclear cells from hemodialysis patients [61]. 1,25(OH)2D3 addition in the culture medium of either monocytes or macrophages induces an up-regulation and a down-regulation of IL-1 and IL-6 mRNA, respectively. These results were abolished or modified when cells, before being stimulated with LPS or TNF-α, were pre-incubated with 1,25(OH)2D3 [62]. 1,25(OH)2D3 triggers a lessened expression of IL-10, followed by an increased production, at a later stage [46].

Such regulation variability may explain some observed discrepancies between the mouse strains used in the current study. Genetically similar mice from the same ages were fed with the same diet, during the same time window, and the same brain areas were analyzed. Yet, one strain differs from the other by carrying two mutated human genes – APP and PSEN1 - that induce molecular, morphological and behavioral changes. The 5XFAD mouse model is an inflammatory model [4, 63] and, as mentioned above, 1,25(OH)2D3 induces a different response when acting on an inflamed tissue. Interestingly, this may even apply to the canonical pathways that are dysregulated in both strains (Additional file 4). In most if not all cases, the two lists of misexpressed genes are not fully identical. This confirms that the pleiotropic action of vitamin D3 and its related metabolites strongly varies according to the health status of the animal.

In addition, the strain-associated differences may be related to the mechanism of action of vitamin D metabolites at play, whether it is a genomic response or the induction of membrane based signaling pathways. In a recent review, Michael Berridge develops the hypothesis that vitamin D acts as a guardian of phenotypic stability in a cell- and context-specific manner [64]. This stability is maintained through modulation of various intracellular signaling pathways that are linked to vitamin D action. For instance, vitamin D is capable of regulating the expression of many components of pathways activated by insulin, TGFB1, WNT and NOTCH [65], whose mRNA expression is affected in our study.

### A strong link with Alzheimer’s disease in both Wt and Tg

The 5XFAD model is based on the insertion of two human transgenes leading to a major increase of Aβ 1-42 that accumulates rapidly in the brains of transgenic animals. Overall, this model is both an inflammatory and amyloidogenic model of Alzheimer’s disease [2, 4, 66]. As seen so far, the inflammatory component present in Tg animals is highly addressed by vitamin D3 treatment and, to a lesser extent, in Wt animals. Other effects of gene dysregulation in the brain of both Wt and Tg mice relate to Alzheimer’s disease. For instance, one of the commonly dysregulated pathways, described in Additional file 4, concerns the neuroprotective role of THOP1. THOP1, an oligopeptidase whose levels increase upon Aβ exposure, is found overexpressed in human AD brains, and appears to engage in neuroprotective activities against toxic effects of Aβ at early stages of the disease [67]. This pathway is significantly affected in our study, however it is difficult to conclude about the outcome induced by such dysregulation at the functional level, especially since the expression of different genes in the pathway is modified according to the genotype.

Within the THOP1 pathway, the expression of angiotensin-converting enzyme (ACE), which converts angiotensin I to angiotensin II, a potent vasoconstrictor, is affected in both wild type and transgenic animals. No study has focused on the effect of vitamin D3 supplementation on ACE expression. However, it has been observed that VDR knockout mice display an increased production of ACE mRNA [68]. Interestingly, this metalloprotease is involved in Alzheimer’s disease since human ACE increases degradation and cleavage of Aβ [69–72].

Another interesting result is the finding that a number of DEGs relate to vascular processes. Angiogenesis is a complex process that involves the activation and modification of several signaling pathways within endothelial cells. A key mediator of this process is VEGF. Pathological angiogenesis is now considered as a key contributor to the development of AD. Substantial evidence suggests that the neurodegenerative process can be caused by cerebral hypoperfusion which induces reduced oxygen, glucose and nutrient supply to the brain, and damages the parenchymal cells and the blood–brain barrier (BBB). BBB dysfunction promotes oxidative stress, inflammation, paracellular permeability and dysregulation of nitric oxide [73]. Several signaling pathways such as VEGF signaling, tight junction signaling, nitric oxide signaling in macrophages and eNos signaling are disturbed by vitamin D3 treatment, especially in Tg mice.

Conversely, we observed a misexpression of *Apoe* mRNA only in wild type supplemented animals. This protein is associated to cardiovascular diseases as well as Alzheimer’s disease [74]. No direct interaction between vitamin D3 and ApoE has been unveiled so far. However, several studies indicate putative interplays. For example, the VDR mRNA regulates the phosphorylation and production of STAT1 [75] which in turn is involved in the expression of apoE mRNA [76]. Furthermore, the vitamin D system interacts with IGF1 and Esr1 (see below), the latter upregulating *Apoe* mRNA in rat primary culture of hippocampal neurons [77]. On the other hand, ApoE ɛ4 alleles have been associated with reduced memory function and higher serum 25(OH)D concentrations [78].

Overall, the fact that a large number of vitamin D-related genes are categorized as AD-related in both Wt and Tg animals is intriguing. This is suggestive of a close link between vitamin D signaling and AD-related signaling, as has been proposed by researchers [25, 79]. Although we observed a positive effect of vitamin D3 treatment on the amount of amyloid plaques, we never detected a misexpression of the endogenous *App* transcript in our transgenic or wild type animals. This comes as a relative surprise since it has been reported that either the cotransfection of VDR or a calcitriol treatment reduces, in a dose dependent manner, APP transcription in neuroblastoma cells [18]. It is hypothesized that VDR interacts with SMAD3 [80], a transcription factor known to regulate APP transcription through TGF beta signaling [81]. However, our data failed to confirm these findings. In wild type mice, vitamin D3 supplementation induces an under-expression of *Smad6, Tgfb1* and *Tgfbr1* transcripts without affecting *App* expression. In addition, no misexpression of these four mRNAs is observed in transgenic animals. It is therefore likely that other intermediate molecules, such as those described below, are involved in the reduction of amyloid burden.

### An unexpected regulation of oxytocin and arginine vasopressin transcripts in the cortex

One very intriguing result is the mRNA regulation of a number of hormones classically known to be present in the pituitary axis. It is true that OXT and AVP, for instance, act as both hormones and central neurotransmitters. However, to our knowledge, the presence of local regulation of gene expression in the hippocampus or cortex for both these hormones has never been reported before. Binding sites for both hormones have been described in different central regions such as the amygdala, septum and olfactory region [82]. However, while AVP receptors have been reported within the hippocampus no OXT receptor has been found in this area [83]. Both neuropeptides regulate rodent, primate and human social behaviors and stress responses [84]. AVP influences memory processes in the brain by facilitating memory consolidation and retrieval [85, 86]. Regarding its role in AD pathology, a significant decrease in AVP concentration was found in the brain of AD patients while application of AVP improves learning and memory in aged people and in rats having received Aβ [87–90]. OXT regulates hippocampal synaptic plasticity and improves hippocampal-dependent cognitive functions [91–93]. The direct or indirect regulation of these neuropeptides by vitamin D3 could have important implications for a number of behavioral and cognitive processes in both the normal and pathological brain. Differential expression of these two genes (*Oxt* and *Avp*) was then measured by qPCR. A significant increase for both these genes was only confirmed in the cortex, although the same tendency is observed in the hippocampus. It appears that important inter-individual variations are present among the tested mice, further supporting the idea that vitamin D3 acts not only in a space- and time-specific manner, but also in an individual-specific way.

### A putative role of insulin growth factor 1 and estrogen receptor 1 as downstream regulators

Four other gene expressions were assessed with qPCR and, once again, not all were validated in each brain area analyzed. *Lcn2* and *Ptgs2* are both involved in inflammatory signaling processes and have been found to contribute to amyloid response in AD [94, 95]. *Esr1* and *Igf1* induce signaling pathways known to be affected in AD. Several interactions between the estrogen and vitamin D endocrine system have been described so far. 1,25(OH)2D3 may play an important role in estrogen biosynthesis suggested by the fact that serum estrogen levels are decreased in VDR knock-out mice as compared with wild-type mice [96]. The direct action of vitamin D on estrogen signaling is also cell-specific as demonstrated by the fact that 1,25-(OH)2D3 increases estrogen receptor (ER) expression in osteoblast-like cells, while it exerts a negative effect on ER in MCF-7 human breast cancer cells [97, 98]. An alteration in ER distribution in AD hippocampal neurons has been described, and, more precisely, a shift in cellular localization of these receptors inhibits the development of AD pathology [99, 100]. Collectively, investigations suggest that levels of estrogen receptors play an important role in neuroprotection and against neuroinflammation-induced degeneration in AD [101, 102]. ESR1 has also been shown to interact directly with amyloid processing through non-genomic signaling via activation of MAPK/ERK pathway [103]. Recently, two potential negative VDREs have been identified in the promoter region of *Esr1* [104] suggesting a direct transcriptional action of vitamin D/VDR on ER leading to an altered estrogen signaling mechanism important to both Wt and AD-like brains. A possible mechanism by which ESR1 confers neuroprotective effects in AD is through interaction with IGF1R. Both in vivo and in vitro studies have revealed interaction between ERs and IGF1R in the promotion of neuronal survival, synaptic plasticity and against Aβ toxicity [105, 106]. Insulin signaling is emerging as an important event in neurodegenerative processes and aging in general. However, inconsistencies regarding the role of insulin-like signaling and its association to AD have appeared. In rodents, reduced insulin/IGF1 signaling slows aging and therefore prevents development of neurodegenerative diseases [107] but normal aging is associated with declining levels of IGF1 and administration of insulin can improve cognitive function in AD patients [108, 109]. Regardless, in vivo evidence shows that AD-like pathology is significantly delayed in IGF1R knock out mouse models of AD [110, 111]. Potential crosstalks between vitamin D and IGF1 signaling have been put forward. VDR knock-out mice display decreased expression of *Igf1, Igf1r* and *Nf- kb* [112]. 1,25(OH)2D3 also regulates the expression of a well-known anti-aging gene, Klotho (*Kl*), whose expression is modified in the hippocampus of both Wt and Tg mice in our study. *Kl* dysregulation is linked to modulation of Insulin/IGF1 signaling pathways [113]. Taken together, these results largely support both direct and indirect action of vitamin D/VDR on ESR1 and IGF1 signaling pathways in both Wt and 5XFAD mice, as a mechanism to reduce brain aging processes.

### Convergent data on amyloid plaque load and divergent results on astrogliosis

The bulk of our study focuses on describing the similarities or differences induced by chronic vitamin D3 supplementation at the transcriptomic level in Wt and Tg animals at M9. Importantly, the altered gene expression translates into a modification of functional outcomes. We note a link between the action of vitamin D3 on gene regulation in our model and its effect at the histological and behavioral levels. We show here that 5 months of vitamin D3 treatment restores learning and memory performance in Tg animals in both the Y-maze and RAWM but does not affect cognitive outcome in Wt mice. This is accompanied by a decrease in the number of amyloid plaques, astrocytic reactivity and subtle changes in microglial activation for Tg animals having been fed a vitamin D3 diet. Several studies have shown an improvement in memory and cognitive function in different models of AD or aging following vitamin D2, vitamin D3 or 1,25(OH)2D3 treatment. In their work, Yu and colleagues used young AβPP transgenic mice and found an amelioration of performance in the Morris Water Maze (MWM) for animals under a high vitamin D3 diet. These behavioral changes were accompanied by a diminished Aβ load, along with an increase in astrocytic reactivity, NGF levels and decreased TNF-α in the brain of treated mice. This study focused on a preventive action of vitamin D3, which might explain some of the differences observed when comparing their data to ours. Specifically, the increase in astrocytic marker GFAP is inconsistent with the diminished GFAP staining observed in our study [26]. This discrepancy might reflect the balance by which vitamin D3 exerts its effects according to the pathological context and the time of delivery. An increase in GFAP expression is a hallmark of reactive gliosis and is found in many neuropathological conditions, including AD. The functional changes associated to this increased expression however are not fully understood. To study the functional outcomes of GFAP dysregulation on the surrounding brain tissue and cells, GFAP knock-outs (KO) have been generated and when combined to brain injury models, studies suggest that GFAP increase is associated to protective mechanisms [114, 115]. When GFAP KO mice were crossed with a mouse model for AD, the deposition of amyloid plaques was accelerated. Furthermore, neuritic dystrophy was exacerbated and the interaction of astrocytes with plaques was decreased [116]. These observations lead to the conclusion that astrogliosis can be considered as a brain defense mechanism attempting to prevent plaque formation and neuronal damage, and this would take place during the onset of the disease. However, we cannot rule out that, at the same time, gliosis affects the synaptic and metabolic support functions of astrocytes [116, 117]. It is then probable that with age and repeated insults, chronic astrocyte reactivity may become deleterious, engaging the opposite effect of vitamin D3 on GFAP expression when compared to a younger tissue. We and others have shown that genes involved in glial reactivity and inflammation are among the earliest affected during disease pathogenesis in the 5xFAD model and that these are correlated with cognitive impairment [4]. We show here that vitamin D3 is able to act specifically on both astrocytic and microglial activation at both the transcriptomic and histological levels. In the AD brain, microglial activation is the driving force for the elaboration of an inflammatory cascade [118] and reports show an upregulation of VDR mRNA in microglia stimulated by Aβ in vitro [119]*,* indicating that microglia found around amyloid plaques could increase their responsiveness to vitamin D metabolites. Microglia, unlike the other cells of the nervous system, stems from the myeloid lineage and is considered the macrophages of the brain. As discussed thoroughly here and elsewhere, the vitamin D system displays potent anti-inflammatory roles in immune cells and the increase in microglial activity in the frontal cortex of 5xFAD mice reflects the capacity of vitamin D3 to balance the pro- and anti-inflammatory cytokine production regulated by microglia [118, 120]. In addition, this enhanced activation of microglia at the proteomic level could also reflect increased phagocytic activity of Aβ, a process demonstrated to be promoted by vitamin D3 in the macrophages of AD patients [121, 122]. Once again, it appears that vitamin D3 acts on both nervous system and the immune system pathways to reduce alteration in neuronal metabolism and further preserve cognitive outcomes.

### An age- and concentration-dependent action on cognition

The study by Yu and colleagues does not present the effect of vitamin D3 on cognitive functions in the Wt counterpart, leaving no comparison possible [26]. Accordingly, an 8 week treatment with intraperitoneal injections of 1,25(OH)2D3 in a different mouse model of AD restored learning and memory deficits, assessed by the fear conditioning paradigm, compared to vehicle-treated Tg mice but did not affect cognitive performance in non-Tg animals [123]. Yet, another study, which tested a 7 months vitamin D2 supplementation in another AD mouse model, concluded that, in this case, vitamin D2 improved cognitive performance more reliably in Wt mice rather than Tg at M9 [124]. And when focusing solely on the implication of vitamin D in normal aging and memory disabilities, Latimer and colleagues showed that a high vitamin D3 diet (10,000 IU/Kg diet) lasting 5 to 6 months prevented cognitive decline in aging rats. The study demonstrates a causal relationship between enhanced learning and memory during aging with vitamin D3 status which is underlined by enhanced synaptic transmission due to gene expression changes in the brain [30]. At approximately the same time, Briones and Darwish performed subcutaneous injections of 1,25(OH)2D3, for 21 days, to both young and aged rats (6 and 20 months, respectively) and observed an attenuation of cognitive impairment accompanied by enhanced brain energy metabolism, modulation of inflammatory and redox state in aged rats after treatment compared to controls [125, 126]. Both these studies underlie a role for vitamin D metabolite supplementation in the preservation of cognitive function in “normal” aging animals. The middle-age period is characterized by the onset of subtle changes in cognitive performance that might not be detected by the straightforward tests that are the Y-maze and RAWM. It is probable that M9 is not old enough to detect memory decline in Wt animals or that tests aiming at detecting more subtle performance changes in executive function or processing speed would be required to allow analysis of vitamin D3 treatment on cognitive performance in Wt aging animals.

### Conclusions

Classically, investigations into physiological processes and neurodegenerative disease pathogenesis have moved from the whole organism to the molecular level as the advent of genome-wide microarrays and large-scale genome sequencing have become powerful tools for probing physiological mechanisms. Vitamin D signaling is ideally suited for genomic analysis as its main receptor VDR is a direct regulator of gene transcription. However, although transcriptomic technologies have evolved greatly, there is still no consensus about an optimal way to analyze transcriptomic data. This renders comparison with other studies rather difficult, especially when platform and data analysis programs differ. The growing knowledge about epigenetic mechanisms at play in gene regulation and its relevance to vitamin D action in the brain could not be addressed in this study but needs further investigation in future experimental designs. The absence of a clear consensus motif for VDREs also impedes the pinpointing of direct vitamin D targets. Moreover, a 5-month supplementation entails that transcriptional activation is not the only mechanism at play, and that membrane-based actions of vitamin D and its related metabolites are to be considered. The interactions between the vitamin D system and other signaling pathways are clearly as important as direct transcriptional activation or repression of 1,25(OH)2D3 targets, as shown in this study.

The aim of this study was to i) assess the potential therapeutic benefit and the mechanisms of action of a chronic vitamin D3 supplementation in an AD model and ii) extract information about specific signaling pathways affected by vitamin D3 in the nervous system. Therefore, validating each DEG obtained by microarray assay using qPCR or proteomic approaches was largely beyond the scope of the study but this needs to be further addressed, especially for those genes put forward as key partners or downstream actors. It is important to note that the discordant results observed between microarray data and qPCR analysis might reflect the importance of inter-individual variability in response to vitamin D3 treatment but also the limitations for each method used. Indeed, transcriptomic data are normalized to global measures while qPCR results are obtained in reference to one housekeeping gene. Concerning *Igf1* mRNA quantification, it is also likely that putatively low levels followed by further reduction in gene expression is more error prone and reaches the limit of qPCR analysis. Moreover, as our study emphasizes the time-, background- and individual-specific action of vitamin D3 in the brain, it is obvious that differential activity at the transcriptomic level is operated between the cortex and hippocampus. Additional analysis of our data would allow for more precise deciphering of vitamin D3 impact in each brain area known to be affected in AD. The study was also designed using a female cohort, and it would seem extremely relevant to repeat such experiments in male animals. This could help contribute to the ongoing debate about the relevance of gender differences in the pathogenesis of the disease. Especially since estrogen signaling appears to play an important part in the events observed here, along with the mRNA dysregulation of a certain number of hormones, we cannot exclude a sex-impact to be considered in the way vitamin D3 acts on brain cells.

Lastly, animal studies using vitamin D treatment in AD models, including ours, show improvement in various markers of the disease but interventional clinical trials, to date, failed to show such effects. It is important to bear in mind that animal models of AD do not recapitulate fully the complex physiopathology of the disease and major differences in regulation by the vitamin D signaling system are observed, according to the species concerned [127], possibly further increasing the gap between the results obtained from animal and human studies. Moreover, we chose to place ourselves in a therapeutic context, however, reproducing such a study in a preventive manner would allow refining the analysis of vitamin D3 action in a time-dependent manner. Indeed, one major current problem concerning clinical trials, using vitamin D as a protective agent against cognitive decline and AD, is the time and dose for treatment. When comparing our study to others, we highlight the differences in functional outcomes depending on the time of treatment, the dose or metabolite used. As with clinical trials, there is a great need for additional work in rodents before deciphering the exact mechanisms of action of vitamin D in the nervous system. However, we feel confident that the present dataset is a useful public resource which reflects the variety and pleiotropy of vitamin D3 actions in the brain with particular emphasis on the crosstalk between vitamin D3 activated pathways and those affected in AD and should allow future studies to further validate the use of vitamin D3 as a potent therapeutic agent in AD.

## Methods

### Animals and experimental design

Female 5XFAD transgenic mice were used for this study. These mice overexpress two transgenes bearing five mutations linked to familial AD: human *APP* (Swedish mutation K670N, M671L; Florida mutation I716V; London mutation V717I) and human *presenilin 1* (*PSEN1* M146L, L286V), under transcriptional control of the mouse Thy1 promoter. 5XFAD lines from the B6/SJL genetic background were maintained by crossing hemizygous transgenic mice with B6/SJL F1 breeders. These mice exhibit AD-related symptoms earlier than other animal models and amyloid deposition starts in the cortex and subiculum at 2 months of age [2, 128]. Heterozygous 5XFAD transgenic animals and wild type (Wt) controls were obtained after breeding of progenitors purchased from the Jackson Laboratory. Newborn pups were genotyped by polymerase chain reaction (PCR) of tail DNA biopsies in order to detect the human *PSEN1* gene as previously described [128]. Animals were weaned at 4 weeks of age and fed an identical diet until M4. Mice were then allocated to different groups: Wt animals on a control diet (1000 IU/kg) (*n* = 10), Wt animals on a vitamin D3 enriched diet (7500 IU/kg) (*n* = 11), transgenic animals on a control diet (*n* = 14) and transgenic animals fed with the vitamin D3 enriched diet (*n* = 16) (INRA, France). Mice were tested at M4, M6 and M8 in the Y-maze, and at M8 in the 6-radial-arm water maze before euthanasia at M9. Animal experiments were approved by the Ethics Committee of the Medical Faculty of Marseille and were carried out in accordance with the guidelines published in the European Communities Council Directive of November 24, 1986 (86/609/EEC). All efforts were made to reduce animal suffering and the number of mice needed for the study.

### Behavioural testing

#### Y-maze

Spontaneous alternation in the Y-maze was tested according to the following protocol: each mouse was placed in a random arm of the symmetrical Y-maze and was allowed to explore freely through the maze during an 8 min session. The sequence and total number of arms entered was recorded. Arm entry was considered complete when the hind paws were completely in the arm. Experiments were done blind with respect to the genotype and diet of the mice. Washing with water and ethanol was performed between each passage. Percentage of alternation was determined as follows: number of triads containing entries into all three arms/maximum possible alternations (total number of arms entered – 2) x 100.

#### 6-Radial arm water maze

The 6-RAWM is a hybrid of the Morris water maze and a radial arm maze. The test allows assessment of spatial and reference memory. The mouse version of this maze uses a circular pool with six swim alleys (arms) radiating out from an open central area with a hidden escape platform submerged at the end of one goal arm. The overall design of the study was adapted from Alamed and collaborators [129], using a 3-day protocol containing 12 sessions per day. Visual cues were displayed around the room and the experimenter remained visible throughout the training sessions. For this procedure the goal arm differed between mice but was held constant for each mouse during all trials with a different start arm on successive trials. The first day of training comprised of alternating between hidden and visible platform. During the 12 trials of the second and third day, the platform remained hidden and the mice were required to use the visual cues to locate the goal arm. When a mouse entered a wrong arm, which did not contain the platform, an error was scored. At the end of the procedure, mice were tested in the circular pool using a visible platform, to ensure their visual abilities were satisfactory for inclusion in the study.

### Tissue isolation

Brain tissues were collected at M9, from all groups. Mice were anesthetized with isoflurane before sacrifice and brains isolated from each animal. The hippocampus and neocortex from a half brain were dissected, snap-frozen in liquid nitrogen and stored at -80 °C until use. The other hemi-brain was placed in 4 % paraformaldehyde during 6 days at 4 °C, for further immunohistological processing.

### Tissue processing and immunostaining

Coronal brain sections from hemi-brains previously collected and fixed in 4 % paraformaldehyde were serially generated using a cryostat and stored at -20 °C in six-well plates containing a cryoprotectant (30 % glycerol; 30 % ethylene glycol (Sigma Aldrich, Saint-Quentin Fallavier, France) diluted in 0,05 M PBS phosphate buffered saline (pH 7.4) until being processed for immunostaining.

After washing in PBS, floating sections were incubated, during 1 h at room temperature (RT), with a blocking buffer (3 % BSA, 0.1 % Triton X-100 in PBS pH 7.4) and, overnight at 4 °C, with the following primary antibodies diluted in the blocking solution: rabbit polyclonal anti-GFAP (1/500, Dako France, Trappes, France), mouse monoclonal anti-Aβ, 6E10 (1/300, Covance, Eurogentec, Angers, France) and rabbit polyclonal anti-IBA1 (1/200, Wako, Sobioda, Montbonnot-Saint-Martin, France). Then, slices were rinsed in PBS and incubated for 90 min at RT with cross-absorbed Alexafluor 488-conjugated anti-rabbit or 594-conjugated anti-mouse secondary antibodies (1/500, Life Technologies, Saint Aubin, France) along with Hoechst blue (1/1000, Sigma Aldrich) in dark conditions. After several washes in PBS, slides were mounted with ProLong Gold Antifade reagent (Life Technologies).

### Microscopic analysis and quantification of gliosis and amyloid plaques

Quantification of gliosis and amyloid plaques were performed on 30 μm thick brain coronal sections co-stained with either GFAP and 6E10 or IBA1 and 6E10 antibodies, at the frontal cortex, hippocampal and sensory cortex levels. Images were acquired using an inverted Axio Observer microscope (Zeiss, Jena, Germany) equipped with DAPI, FITC and TRITC epifluorescence filters. Images of large brain sections were obtained using the mosaic mode of the Axiovision software (Zeiss). For 6E10, GFAP and IBA1 staining quantifications, ImageJ software was used. The pictures were binarized to 16 bit black and white images and a fixed intensity threshold was applied for all images. Percentage of covered area by the fluorescent staining was calculated. Eight mice per group and three sections per mouse at three different levels were used.

### RNA isolation

Total RNA was isolated from the snap-frozen hippocampi and cortices using RNeasy Mini kit (Qiagen, Courtaboeuf, France), according to the manufacturer’s instructions. RNA concentration was determined using a Nanodrop 2000 spectrophotometer (Life Technologies ThermoFisher Scientific, Villebon sur Yvette, France) and RNA integrity assessed on an Agilent 2100 Bioanalyzer (Agilent Technologies, Les Ulis, France).

### Microarray assay

RNA samples from three animals in each group were pooled for microarray hybridization. Sample amplification, labeling, and hybridization were performed in line with the Agilent one-color microarray-base analysis (low input quick amp labeling) protocol (Agilent Technologies). Briefly, total RNA was reverse-transcribed into cDNA using the T7 promoter primer. The reaction intending to synthesize cyanine-3-labeled cRNA from cDNA was performed in a solution containing dNTP mix, T7 RNA polymerase and cyanine 3-dCTP and then incubated at 40 °C for 2 h. Labeled cRNA was purified and fragmented before hybridization on Agilent 8x60k Mouse Gene Expression Arrays (Agilent Technologies), containing 62,975 oligonucleotide probes, at 65 °C for 17 h. Raw microarray signals were scanned and extracted using Agilent Feature Extraction Software (Agilent Technologies). AgiND R package was used for quality control and normalization. Quantile methods and a background correction were applied for data normalization. Microarray data are available in the ArrayExpress database under accession number E-MTAB-1937.

### Microarray data analysis

Biological interpretation of the data was performed using two different programs. The NIH DAVID tool was used to obtain functional annotation clusters related to the datasets of interest. Ingenuity Pathway Analysis (IPA, Ingenuity Systems) was also used to identify biological functions and upstream regulators relevant to the lists of DEGs analyzed. The main criterion to validate a differentially expressed gene was a fold change over 1.5 or under -1.5 when considering expression values in the group supplemented with vitamin D3 relative to the control diet group, whether considering wild type or transgenic animals. Upregulated and downregulated genes were analyzed in the same datasets to obtain the biologically relevant function categories. Right-tailed Fisher’s exact test was used to calculate a *p*-value determining the top statistically significant biological functions assigned to the data set.

### Real-time quantitative PCR (qPCR)

Total RNA (750 ng) was subjected to reverse transcription reaction to synthetize cDNA using oligo dT, RNase Out and M-MLV RT enzyme (Life Technologies, ThermoFisher Scientific) according to the manufacturer’s instructions.

Real time qPCR experiments were carried out with the 7500 Fast real-Time PCR system (Applied Biosystems, ThermoFisher Scientific), using TaqMan® Fast Universal PCR Master Mix (2X) and the following TaqMan® Gene Expression Assays: *Oxt* (Mm00726655_s1), *Avp* (Mm00437761_g1), *Lcn2* (Mm01324470_m1), *Ptgs2* (Mm00478374_m1), *Esr1* (Mm00433149_m1), *Igf1* (Mm00439560_m1), *Gapdh* (Mm99999915_g1). Experiments used 7.5 ng of previously prepared cDNA and samples were run in triplicates on six different biological samples for each group. Relative expression levels were determined according to the ΔΔCt method where the expression level of the mRNA of interest is given by 2^-ΔΔCT^ where ΔΔCT = ΔCT target mRNA − ΔCT reference mRNA (*Gapdh*) in the same sample as previously described [4, 128].

### Statistical analysis

Significant differences between groups were determined using the Kaleida Graph software. We used one-way ANOVA followed by a post-hoc Bonferroni test for multiple comparisons. Kruskal-Wallis test was used to compare two experimental groups. Values represent the mean ± SEM of the indicated number of independent experiments/animals, and the level of significance was set for *p* < 0.05 * or *p* < 0,01**.

## Additional files

Additional file 1: Table S1. List of vitamin D-related transcripts dysregulated in transgenic 5XFAD mice. Dysregulated transcripts in the adult brain of 9 month-old 5XFAD transgenic female mice in comparison with wild type mice. The acronym and the full name of each gene are indicated. Genes with a VDRE are highlighted in grey. (PDF 117 kb) Additional file 2: Comparative analysis of DEGs between different conditions. Table S2. Common DEGs between cortex and hippocampus in Wt mice supplemented with vitamin D3. Table S3. Specific DEGs in cortex of Wt mice supplemented with vitamin D3. Table S4. Specific DEGs in hippocampus of Wt mice supplemented with vitamin D3. Table S5. Common DEGs between cortex and hippocampus in Tg mice supplemented with vitamin D3. Table S6. Specific DEGs in cortex of Tg mice supplemented with vitamin D3. Table S7. Specific DEGs in hippocampus of Tg mice supplemented with vitamin D3. Table S8. Common DEGs between cortex and hippocampus of both Wt and Tg mice supplemented with vitamin D3. (XLSX 214 kb) Additional file 3: Table S9. Common DEGs in at least 3 out 4 conditions (cortex; hippocampus; WtVitD; TgVitD). (XLSX 61 kb) Additional file 4: List of canonical pathways modified by a vitamin D3-enriched diet in Wt and Tg animals. Table S10. Common canonical pathways between Wt and Tg mice supplemented with vitamin D3 and associated DEGs. Table S11. Canonical pathways specifically modified in Wt VitD animals. Table S12. Canonical pathways specifically modified in Tg VitD animals. (XLSX 67 kb) Additional file 5: List of DEGs known to be related to vitamin D action in the corertx and hippocampus of Wt and Tg mice. Genes containing a VDRE sequence are highlighted in grey. Table S13. Vitamin D related DEGs in Tg Cont, Tg VitD and Wt VitD mice. Table S14. Common vitamin D related DEGS between Tg Cont and TgVitD or between Tg Cont and Wt VitD. Table S15. Common vitamin D related DEGs between Tg Cont, Tg VitD and Wt VitD mice. (XLSX 95 kb) Additional file 6: Figure S1. Comparison of the different DEGs affected in the Wnt/β-catenin signaling pathway according to mouse genotype (Wt VitD versus Tg VitD). (PDF 219 kb)

## Abbreviations

1,25(OH)2D3: 1,25-dihydroxycholecalciferol or calcitriol
25(OH)D: 25-hydroxyvitamin D or calcidiol
5XFAD: 5 mutations of familial Alzheimer’s disease
AD: Alzheimer’s disease
APP: amyloid precursor protein
Aβ: amyloid beta peptide
BBB: blood brain barrier
DEG: differentially expressed genes
ESR1: estrogen receptor 1
FC: fold change
IGF1: insulin growth factor 1
IPA: ingenuity pathway analysis
KO: knock-out
M4; M6; M9: month 4; month 6; month 9
MWM: Morris water maze
PSEN1: presenilin 1
RAWM: radial arm water maze
RXR: retinoid X receptor
TG: transgenic
VDR: vitamin D receptor
VDRE: vitamin D responsive element
VitD: vitamin D
WT: wild type
