# Supplementary material for: Vitamin D interacts with Esr1 and Igf1 to regulate molecular pathways relevant to Alzheimer’s disease
Source: Mol Neurodegener. 2016 Mar 1;11:22. doi: 10.1186/s13024-016-0087-2 (PMC4774101; doi:10.1186/s13024-016-0087-2)
Supplement: Additional file 1: — Table S1. List of vitamin D-related transcripts dysregulated in transgenic 5XFAD mice. Dysregulated transcripts in the adult brain of 9 month-old 5XFAD transgenic female mice in comparison with wild type mice. The acronym and the full name of each gene are indicated. Genes with a VDRE are highlighted in grey. (PDF 117 kb) [file 13024_2016_87_MOESM1_ESM.pdf]

Supplementary table 1. List of vitamin D-related transcripts dysregulated in transgenic 5XFAD mice

Gene Symbol	Gene Entrez Name
AASS	aminoadipate-semialdehyde synthase
ABCA1	ATP-binding cassette, sub-family A (ABC1), member 1
ABCC3	ATP-binding cassette, sub-family C (CFTR/MRP), member 3
ABCD2	ATP-binding cassette, sub-family D (ALD), member 2
ACAA2	acetyl-CoA acyltransferase 2
ACACA	acetyl-CoA carboxylase alpha
ACAT2	acetyl-CoA acetyltransferase 2
ACSL1	acyl-CoA synthetase long-chain family member 1
ACSL4	acyl-CoA synthetase long-chain family member 4
ACTA1	actin, alpha 1, skeletal muscle
ACTA2	actin, alpha 2, smooth muscle, aorta
ACVRL1	activin A receptor type II-like 1
ADA	adenosine deaminase
ADAM17	ADAM metallopeptidase domain 17
ADAMTS1	ADAM metallopeptidase with thrombospondin type 1 motif, 1
ADAMTS9	ADAM metallopeptidase with thrombospondin type 1 motif, 9
ADRA1B	adrenoceptor alpha 1B
ADRA1D	adrenoceptor alpha 1D
ADRB3	adrenoceptor beta 3
AEBP1	AE binding protein 1
AFP	alpha-fetoprotein
AGPAT2	1-acylglycerol-3-phosphate O-acyltransferase 2
AGT	angiotensinogen (serpin peptidase inhibitor, clade A, member 8)
AHCY	adenosylhomocysteinase
AHR	aryl hydrocarbon receptor
AHRR	aryl-hydrocarbon receptor repressor
AK2	adenylate kinase 2
AKR1B10	aldo-keto reductase family 1, member B10 (aldose reductase)
ALDH1A3	aldehyde dehydrogenase 1 family, member A3
ALDOA	aldolase A, fructose-bisphosphate
ALDOB	aldolase B, fructose-bisphosphate
ALOX5	arachidonate 5-lipoxygenase
ALOX5AP	arachidonate 5-lipoxygenase-activating protein
ALPL	alkaline phosphatase, liver/bone/kidney
AMPH	amphiphysin
ANTXR1	anthrax toxin receptor 1
ANXA1	annexin A1
ANXA2	annexin A2
APLN	apelin
APOA2	apolipoprotein A-II
APOBEC3B	apolipoprotein B mRNA editing enzyme, catalytic polypeptide-like 3B
APOC2	apolipoprotein C-II
APOE	apolipoprotein E

AQP5	aquaporin 5
AR	androgen receptor
ARHGAP1	Rho GTPase activating protein 1
ARHGDIB	Rho GDP dissociation inhibitor (GDI) beta
ARID3A	AT rich interactive domain 3A (BRIGHT-like)
ARL4C	ADP-ribosylation factor-like 4C
ARNT2	aryl-hydrocarbon receptor nuclear translocator 2
ARTN	artemin
ASPM	asp (abnormal spindle) homolog, microcephaly associated (Drosophila)
ATF3	activating transcription factor 3
ATG9A	autophagy related 9A
ATM	ATM serine/threonine kinase
ATP5B	ATP synthase, H+ transporting, mitochondrial F1 complex, beta polypeptide
ATP7A	ATPase, Cu++ transporting, alpha polypeptide
AURKB	aurora kinase B
AVP	arginine vasopressin
AXIN2	axin 2
AXL	AXL receptor tyrosine kinase
B2M	beta-2-microglobulin
B4GALT1	UDP-Gal:betaGlcNAc beta 1,4- galactosyltransferase, polypeptide 1
BAK1	BCL2-antagonist/killer 1
BATF	basic leucine zipper transcription factor, ATF-like
BATF2	basic leucine zipper transcription factor, ATF-like 2
BCL11A	B-cell CLL/lymphoma 3
BCL2A1	B-cell CLL/lymphoma 11A (zinc finger protein)
BCL3	BCL2-related protein A1
BDNF	brain-derived neurotrophic factor
Bex1	brain expressed gene 1
BHMT	betaine--homocysteine S-methyltransferase
BIRC5	baculoviral IAP repeat containing 5
BLNK	B-cell linker
BMF	Bcl2 modifying factor
BMP4	bone morphogenetic protein 4
BMP6	bone morphogenetic protein 6
BNIP3	BCL2/adenovirus E1B 19kDa interacting protein 3
C1QA	complement component 1, q subcomponent, A chain
C1QB	complement component 1, q subcomponent, B chain
C1R	complement component 1, r subcomponent
C3AR1	complement component 3a receptor 1
C4A/C4B	complement component 4B (Chido blood group)
C9orf9	chromosome 9 open reading frame 9
CA9	carbonic anhydrase IX
CABIN1	calcineurin binding protein 1
CACNA1S	calcium channel, voltage-dependent, L type, alpha 1S subunit
CAMP	cathelicidin antimicrobial peptide

CAPN2	calpain 2, (m/II) large subunit
CASP4	caspase 4, apoptosis-related cysteine peptidase
CASP8	caspase 8, apoptosis-related cysteine peptidase
CAT	catalase
CAV2	caveolin 2
CCK	cholecystokinin
CCL17	chemokine (C-C motif) ligand 17
CCL2	chemokine (C-C motif) ligand 2
CCL3L3	chemokine (C-C motif) ligand 3-like 3
CCL4	chemokine (C-C motif) ligand 4
CCL5	chemokine (C-C motif) ligand 5
Ccl6	chemokine (C-C motif) ligand 6
Ccl7	chemokine (C-C motif) ligand 7
Ccl8	chemokine (C-C motif) ligand 8
Ccl9	chemokine (C-C motif) ligand 9
CCNC	cyclin C
CCNG2	cyclin G2
CCNH	cyclin H
CCNT1	cyclin T1
CCRN4L	CCR4 carbon catabolite repression 4-like (S. cerevisiae)
CD14	CD14 molecule
CD1D	CD1d molecule
CD244	CD244 molecule, natural killer cell receptor 2B4
CD274	CD274 molecule
CD3G	CD3g molecule, gamma (CD3-TCR complex)
CD40LG	CD40 ligand
CD44	CD44 molecule (Indian blood group)
CD55	CD55 molecule, decay accelerating factor for complement (Cromer blood group)
CD63	CD63 molecule
CD68	CD68 molecule
CD74	CD74 molecule, major histocompatibility complex, class II invariant chain
CD80	CD80 molecule
CD82	CD82 molecule
CD83	CD83 molecule
CD86	CD86 molecule
CD9	CD9 molecule
CDH1	cadherin 1, type 1, E-cadherin (epithelial)
CDKN1A	cyclin-dependent kinase inhibitor 1A (p21, Cip1)
Cdkn1c	cyclin-dependent kinase inhibitor 1C (P57)
CDKN2A	cyclin-dependent kinase inhibitor 2A
CDON	cell adhesion associated, oncogene regulated
CEBPA	CCAAT/enhancer binding protein (C/EBP), alpha
CEBPD	CCAAT/enhancer binding protein (C/EBP), delta
CENPA	centromere protein A
CFH	complement factor H

CFI	complement factor I
CFTR	cystic fibrosis transmembrane conductance regulator (ATP-binding cassette sub-family C, member 7)
CH25H	cholesterol 25-hydroxylase
CHAT	choline O-acetyltransferase
CHGA	chromogranin A
CHL1	cell adhesion molecule L1-like
CHRD	chordin
CHRM3	cholinergic receptor, muscarinic 3
CHRNA2	cholinergic receptor, nicotinic, alpha 2 (neuronal)
CHRNB4	cholinergic receptor, nicotinic, beta 4 (neuronal)
CISH	cytokine inducible SH2-containing protein
CLDN2	claudin 2
CLIC5	chloride intracellular channel 5
CMPK2	cytidine monophosphate (UMP-CMP) kinase 2, mitochondrial
CNGA2	cyclic nucleotide gated channel alpha 2
CNOT4	CCR4-NOT transcription complex, subunit 4
COBL	cordon-bleu WH2 repeat protein
COL18A1	collagen, type XVIII, alpha 1
COL1A2	collagen, type I, alpha 2
COL6A3	collagen, type VI, alpha 3
CPB2	carboxypeptidase B2 (plasma)
CPLX2	complexin 2
CPT1A	carnitine palmitoyltransferase 2
CPT2	carnitine palmitoyltransferase 1A (liver)
CREB1	cAMP responsive element binding protein 1
CREM	cAMP responsive element modulator
CRHR1	corticotropin releasing hormone receptor 1
CRP	C-reactive protein, pentraxin-related
CRYAB	crystallin, alpha B
CRYBB1	crystallin, beta B1
CSF1	colony stimulating factor 1 (macrophage)
CSF1R	colony stimulating factor 1 receptor
CSF2	colony stimulating factor 2 (granulocyte-macrophage)
CSHL1	chorionic somatomammotropin hormone-like 1
CSK	c-src tyrosine kinase
CST7	cystatin F (leukocystatin)
CSTA	cystatin A (stefin A)
CTF1	cardiotrophin 1
CTSB	cathepsin B
CTSC	cathepsin C
CTSD	cathepsin D
CTSE	cathepsin E
CTSH	cathepsin H
CTSK	cathepsin K
CTSS	cathepsin S

CTSZ	cathepsin Z
CXCL10	chemokine (C-X-C motif) ligand 10
Cxcl11	chemokine (C-X-C motif) ligand 11
CXCL12	chemokine (C-X-C motif) ligand 12
CXCL13	chemokine (C-X-C motif) ligand 13
CXCL16	chemokine (C-X-C motif) ligand 16
CXCL6	chemokine (C-X-C motif) ligand 6
Cxcl9	chemokine (C-X-C motif) ligand 9
CYBA	cytochrome b-245, alpha polypeptide
CYBB	cytochrome b-245, beta polypeptide
CYBRD1	cytochrome b reductase 1
CYC1	cytochrome c-1
CYCS	cytochrome c, somatic
CYLD	cylindromatosis (turban tumor syndrome)
CYP17A1	cytochrome P450, family 17, subfamily A, polypeptide 1
CYP1B1	cytochrome P450, family 1, subfamily B, polypeptide 1
CYP2A6 (includes others)	cytochrome P450, family 2, subfamily A, polypeptide 6
Cyp2c40 (includes others)	cytochrome P450, family 2, subfamily C, polypeptide 9
CYP2C9	cytochrome P450, family 2, subfamily c, polypeptide 40
Cyp2d9 (includes others)	cytochrome P450, family 2, subfamily d, polypeptide 9
CYP2J2	cytochrome P450, family 2, subfamily J, polypeptide 2
CYP4A11	cytochrome P450, family 4, subfamily A, polypeptide 11
DAB2	Dab, mitogen-responsive phosphoprotein, homolog 2 (Drosophila)
DARS	aspartyl-tRNA synthetase
DAXX	death-domain associated protein
DBH	dopamine beta-hydroxylase (dopamine beta-monooxygenase)
DBI	diazepam binding inhibitor (GABA receptor modulator, acyl-CoA binding protein)
DDX3X	DEAD (Asp-Glu-Ala-Asp) box helicase 5
DDX5	DEAD (Asp-Glu-Ala-Asp) box helicase 3, X-linked
DECR2	2,4-dienoyl CoA reductase 2, peroxisomal
DHRS1	dehydrogenase/reductase (SDR family) member 1
DLAT	dihydrolipoamide S-acetyltransferase
DLC1	DLC1 Rho GTPase activating protein
DLEC1	deleted in lung and esophageal cancer 1
DLK1	delta-like 1 homolog (Drosophila)
DLX3	distal-less homeobox 3
DMTF1	cyclin D binding myb-like transcription factor 1
DNAJC21	DnaJ (Hsp40) homolog, subfamily C, member 21
DOK5	docking protein 5
DTX3	deltex 3, E3 ubiquitin ligase
DUSP2	dual specificity phosphatase 2
DUSP4	dual specificity phosphatase 4
DVL3	dishevelled segment polarity protein 3
DYRK1B	dual-specificity tyrosine-(Y)-phosphorylation regulated kinase 1B
EDN1	endothelin 1

EDN2	endothelin 2
EFNA1	ephrin-A1
EGF	epidermal growth factor
EGLN1	egl-9 family hypoxia-inducible factor 1
EGR1	early growth response 1
EGR2	early growth response 2
EHD4	EH-domain containing 4
EHHADH	enoyl-CoA, hydratase/3-hydroxyacyl CoA dehydrogenase
EIF5A2	eukaryotic translation initiation factor 5A2
ENPP1	ectonucleotide pyrophosphatase/phosphodiesterase 1
ENPP2	ectonucleotide pyrophosphatase/phosphodiesterase 2
EOMES	eomesodermin
EPAS1	endothelial PAS domain protein 1
EPHA7	EPH receptor A7
EPHX1	epoxide hydrolase 1, microsomal (xenobiotic)
EPHX2	epoxide hydrolase 2, cytoplasmic
ERBB4	erb-b2 receptor tyrosine kinase 4
ERRFI1	ERBB receptor feedback inhibitor 1
ESR1	estrogen receptor 1
ESR2	estrogen receptor 2 (ER beta)
EZR	ezrin
F10	coagulation factor VIII, procoagulant component
F11R	coagulation factor X
F8	F11 receptor
FAM132A	family with sequence similarity 132, member A
FAM26F	family with sequence similarity 26, member F
FANCD2	Fanconi anemia, complementation group D2
FAS	Fas cell surface death receptor
FBLN5	fibulin 5
FCER1G	Fc fragment of IgE, high affinity I, receptor for; gamma polypeptide
FCGR1A	Fc fragment of IgG, high affinity Ia, receptor (CD64)
FCGR2B	Fc fragment of IgG, low affinity IIb, receptor (CD32)
FERMT2	fermitin family member 2
FES	FES proto-oncogene, tyrosine kinase
FFAR2	free fatty acid receptor 2
FGF10	fibroblast growth factor 2 (basic)
FGF2	fibroblast growth factor 10
FGF23	fibroblast growth factor 23
FGFR2	fibroblast growth factor receptor 2
FGFR4	fibroblast growth factor receptor 4
FGL2	fibrinogen-like 2
FHL3	four and a half LIM domains 3
FLNA	filamin A, alpha
FLNC	filamin C, gamma
FMN2	formin 2
FOS	FBJ murine osteosarcoma viral oncogene homolog

FOSB	FBJ murine osteosarcoma viral oncogene homolog B
FOSL2	FOS-like antigen 2
FOXC1	forkhead box C1
FOXC2	forkhead box C2
FTL	ferritin, light polypeptide
FUCA1	fucosidase, alpha-L- 1, tissue
FUT7	fucosyltransferase 7 (alpha (1,3) fucosyltransferase)
GAD1	glutamate decarboxylase 1 (brain, 67kDa)
GAPDH	glyceraldehyde-3-phosphate dehydrogenase
GAS1	growth arrest-specific 1
GATA4	GATA binding protein 4
GBE1	glucan (1,4-alpha-), branching enzyme 1
GBP2	guanylate binding protein 2, interferon-inducible
GBP5	guanylate binding protein 5
GBP6	guanylate binding protein family, member 6
Gcg	glucagon
GCHFR	GTP cyclohydrolase I feedback regulator
GCK	glucokinase (hexokinase 4)
GDI1	GDP dissociation inhibitor 1
GFAP	glial fibrillary acidic protein
GFPT2	glutamine-fructose-6-phosphate transaminase 2
GIGYF1	GRB10 interacting GYF protein 1
GJD2	gap junction protein, delta 2, 36kDa
GLIPR1	GLI pathogenesis-related 1
GLS	glutaminase
Gm21596/Hmgb1	high mobility group box 1
GNAI2	guanine nucleotide binding protein (G protein), alpha inhibiting activity polypeptide 2
GNAO1	guanine nucleotide binding protein (G protein), alpha activating activity polypeptide O
GOT1	glutamic-oxaloacetic transaminase 1, soluble
GP1BB	glycoprotein Ib (platelet), beta polypeptide
GRIA2	glutamate receptor, ionotropic, AMPA 2
GRIN1	glutamate receptor, ionotropic, N-methyl D-aspartate 1
GRM1	glutamate receptor, metabotropic 1
GRN	granulin
GSTA5	glutathione S-transferase alpha 5
GSTM3	glutathione S-transferase mu 3 (brain)
HADHA	hydroxyacyl-CoA dehydrogenase/3-ketoacyl-CoA thiolase/enoyl-CoA hydratase (trifunctional protein), alpha subunit
HAS2	hyaluronan synthase 2
HCK	HCK proto-oncogene, Src family tyrosine kinase
HDAC9	histone deacetylase 9
HDGF	hepatoma-derived growth factor
HIST2H2AA3/HIST2H2AA4	histone cluster 2, H2aa3
HIST4H4	histone cluster 4, H4

HIVEP1	human immunodeficiency virus type I enhancer binding protein 1
HK2	hexokinase 2
HLA-A	major histocompatibility complex, class I, A
HLA-DMB	major histocompatibility complex, class II, DM beta
HLA-DQA1	major histocompatibility complex, class II, DQ alpha 1
HLA-DRB5	major histocompatibility complex, class II, DR beta 5
HLA-E	major histocompatibility complex, class I, E
HLA-G	major histocompatibility complex, class I, G
HMBS	hydroxymethylbilane synthase
HMGA1	high mobility group AT-hook 1
HMGCS2	3-hydroxy-3-methylglutaryl-CoA synthase 2 (mitochondrial)
HMOX1	heme oxygenase 1
HNRNPA2B1	heterogeneous nuclear ribonucleoprotein A3
Hnrnpa3	heterogeneous nuclear ribonucleoprotein A2/B1
HOMER1	homer scaffolding protein 1
HOXB1	homeobox B1
HPSE	heparanase
HSD11B1	hydroxysteroid (11-beta) dehydrogenase 1
HSPA4	heat shock 70kDa protein 4
HSPA8	heat shock 70kDa protein 8
HSPB1	heat shock 27kDa protein 1
ICOSLG/LOC102723996	inducible T-cell co-stimulator ligand
IFI16	interferon, gamma-inducible protein 16
Ifi202b	interferon activated gene 202B
IFI35	interferon-induced protein 35
Ifi47	interferon gamma inducible protein 47
IFIH1	interferon induced with helicase C domain 1
IFIT1	interferon-induced protein with tetratricopeptide repeats 1
IFIT1B	interferon-induced protein with tetratricopeptide repeats 1B
IFIT3	interferon-induced protein with tetratricopeptide repeats 3
IFITM3	interferon induced transmembrane protein 3
IFNA4	interferon, alpha 4
IFNAR2	interferon (alpha, beta and omega) receptor 2
IFNG	interferon, gamma
IFNGR1	interferon gamma receptor 1
IGF1	insulin-like growth factor 1 (somatomedin C)
IGF1R	insulin-like growth factor 1 receptor
IGF2	insulin-like growth factor 2
IGFBP2	insulin-like growth factor binding protein 2, 36kDa
IGFBP5	insulin-like growth factor binding protein 5
Igtp	interferon gamma induced GTPase
IKZF2	IKAROS family zinc finger 2 (Helios)
IL10RA	interleukin 10 receptor, alpha
IL13RA2	interleukin 13 receptor, alpha 2
IL16	interleukin 16
IL17RA	interleukin 17 receptor A

IL1B	interleukin 1, beta
IL1RL1	interleukin 1 receptor-like 1
IL21R	interleukin 21 receptor
IL33	interleukin 33
IL3RA	interleukin 3 receptor, alpha (low affinity)
IL4R	interleukin 4 receptor
IL6R	interleukin 6 receptor
IL7R	interleukin 7 receptor
IL9R	interleukin 9 receptor
INHBA	inhibin, beta A
INMT	indolethylamine N-methyltransferase
INPP5D	inositol polyphosphate-5-phosphatase, 145kDa
INSIG2	insulin induced gene 2
INSR	insulin receptor
INSRR	insulin receptor-related receptor
IRF1	interferon regulatory factor 1
IRF5	interferon regulatory factor 5
IRF6	interferon regulatory factor 6
IRF8	interferon regulatory factor 8
IRF9	interferon regulatory factor 9
Irgm1	immunity-related GTPase family M member 1
IRS1	insulin receptor substrate 1
IRX1	iroquois homeobox 1
IRX3	iroquois homeobox 3
ISG15	ISG15 ubiquitin-like modifier
ITGA1	integrin, alpha 1
ITGAM	integrin, alpha M (complement component 3 receptor 3 subunit)
ITGAX	integrin, alpha X (complement component 3 receptor 4 subunit)
ITGB2	integrin, beta 2 (complement component 3 receptor 3 and 4 subunit)
ITGB3	integrin, beta 3 (platelet glycoprotein IIIa, antigen CD61)
ITGB5	integrin, beta 5
ITGB8	integrin, beta 8
JRK	Jrk homolog (mouse)
JUN	Jun proto-oncogene
KAT2B	K(lysine) acetyltransferase 2B
KCND3	potassium channel, voltage gated Shal related subfamily D, member 3
KCNJ3	potassium channel, inwardly rectifying subfamily J, member 3
KCNQ2	potassium channel, voltage gated KQT-like subfamily Q, member 2
KDR	kinase insert domain receptor
KHSRP	KH-type splicing regulatory protein
KIF1A	kinesin family member 1A
KIRREL2	kin of IRRE like 2 (Drosophila)
KL	klotho
KLF10	Kruppel-like factor 10
KLF5	Kruppel-like factor 5 (intestinal)
KLK3	kallikrein-related peptidase 3

KLK6	kallikrein-related peptidase 6
KRT14	keratin 14, type I
KRT18	keratin 18, type I
KRT8	keratin 8, type II
L1CAM	L1 cell adhesion molecule
LAMA3	laminin, alpha 3
LAMP2	lysosomal-associated membrane protein 2
LBP	lipopolysaccharide binding protein
LCN2	lipocalin 2
LCP2	lymphocyte cytosolic protein 2 (SH2 domain containing leukocyte protein of 76kDa)
LDHA	lactate dehydrogenase A
LDLR	low density lipoprotein receptor
LEPR	leptin receptor
LGALS12	lectin, galactoside-binding, soluble, 12
LGALS3	lectin, galactoside-binding, soluble, 3
LGALS3BP	lectin, galactoside-binding, soluble, 3 binding protein
LGR5	leucine-rich repeat containing G protein-coupled receptor 5
LILRB4	leukocyte immunoglobulin-like receptor, subfamily B (with TM and ITIM domains), member 4
LIPA	lipase A, lysosomal acid, cholesterol esterase
LITAF	lipopolysaccharide-induced TNF factor
LNPEP	leucyl/cystinyl aminopeptidase
LPL	lipoprotein lipase
LSP1	lymphocyte-specific protein 1
LSS	lanosterol synthase (2,3-oxidosqualene-lanosterol cyclase)
LTBR	lymphotoxin beta receptor (TNFR superfamily, member 3)
LTC4S	leukotriene C4 synthase
LUM	lumican
LYN	LYN proto-oncogene, Src family tyrosine kinase
LYZ	lysozyme
MAD2L1	MAD2 mitotic arrest deficient-like 1 (yeast)
MAFB	v-maf avian musculoaponeurotic fibrosarcoma oncogene homolog B
MAFF	v-maf avian musculoaponeurotic fibrosarcoma oncogene homolog F
MALL	mal, T-cell differentiation protein-like
MAOA	monoamine oxidase A
MAP3K8	mitogen-activated protein kinase kinase kinase 8
MAPK1	mitogen-activated protein kinase 1
MASP2	mannan-binding lectin serine peptidase 2
MAZ	MYC-associated zinc finger protein (purine-binding transcription factor)
MBOAT2	membrane bound O-acyltransferase domain containing 2
MCF2L	MCF.2 cell line derived transforming sequence-like
MCL1	myeloid cell leukemia 1
MCM3	minichromosome maintenance complex component 3
MEF2C	myocyte enhancer factor 2C
MEOX1	mesenchyme homeobox 1

MFGE8	milk fat globule-EGF factor 8 protein
MGEA5	meningioma expressed antigen 5 (hyaluronidase)
MGMT	O-6-methylguanine-DNA methyltransferase
MGP	matrix Gla protein
MITF	microphthalmia-associated transcription factor
MKI67	marker of proliferation Ki-67
MLXIPL	MLX interacting protein-like
MLYCD	malonyl-CoA decarboxylase
MMP11	matrix metallopeptidase 11
MMP9	matrix metallopeptidase 9
MPG	N-methylpurine-DNA glycosylase
MPL	MPL proto-oncogene, thrombopoietin receptor
MPO	myeloperoxidase
MR1	major histocompatibility complex, class I-related
MRAS	muscle RAS oncogene homolog
MSH5	mutS homolog 5
MSR1	macrophage scavenger receptor 1
MST1R	macrophage stimulating 1 receptor
Mt1	metallothionein 1
Mt2	metallothionein 2
MTCH2	mitochondrial carrier 2
Mx1/Mx2	MX dynamin-like GTPase 1
MYBL2	v-myb avian myeloblastosis viral oncogene homolog-like 2
MYCN	v-myc avian myelocytomatosis viral oncogene neuroblastoma derived homolog
MYH2	myosin, heavy chain 2, skeletal muscle, adult
MYH8	myosin, heavy chain 8, skeletal muscle, perinatal
MYL7	myosin, light chain 7, regulatory
MYLK	myosin light chain kinase
MYOD1	myogenic differentiation 1
NAE1	NEDD8 activating enzyme E1 subunit 1
NAGLU	N-acetylglucosaminidase, alpha
NANOG	Nanog homeobox
NAV2	neuron navigator 2
NCF1	neutrophil cytosolic factor 1
NCF2	neutrophil cytosolic factor 2
NCOA7	nuclear receptor coactivator 7
NDRG4	NDRG family member 4
NET1	neuroepithelial cell transforming 1
NEURL3	neuralized E3 ubiquitin protein ligase 3
NF2	neurofibromin 2 (merlin)
NFATC1	nuclear factor of activated T-cells, cytoplasmic, calcineurin-dependent 1
NFE2L2	nuclear factor, erythroid 2-like 2
NFKB1	nuclear factor of kappa light polypeptide gene enhancer in B-cells 1
NFKBIB	nuclear factor of kappa light polypeptide gene enhancer in B-cells inhibitor, beta

NGF	nerve growth factor (beta polypeptide)
NID1	nidogen 1
NOS2	nitric oxide synthase 2, inducible
NOS3	nitric oxide synthase 3 (endothelial cell)
NOTCH1	notch 1
NOTCH3	notch 3
NPC1L1	NPC1-like 1
NPPA	natriuretic peptide A
NPY	neuropeptide Y
NR1D1	nuclear receptor subfamily 1, group D, member 1
NR1I3	nuclear receptor subfamily 1, group I, member 3
NR2C1	nuclear receptor subfamily 2, group C, member 1
NR3C1	nuclear receptor subfamily 3, group C, member 1 (glucocorticoid receptor)
NR4A1	nuclear receptor subfamily 4, group A, member 1
NR4A2	nuclear receptor subfamily 4, group A, member 2
NR4A3	nuclear receptor subfamily 4, group A, member 3
NRAP	nebulin-related anchoring protein
NRARP	NOTCH-regulated ankyrin repeat protein
NRN1	neuritin 1
NSF	N-ethylmaleimide-sensitive factor
NT5E	5'-nucleotidase, ecto (CD73)
NTS	neurotensin
OAS1	2'-5'-oligoadenylate synthetase 1, 40/46kDa
OAS2	2'-5'-oligoadenylate synthetase 2, 69/71kDa
OASL	2'-5'-oligoadenylate synthetase-like
OGN	osteoglycin
Olfr1508	olfactory receptor 1508
OMP	olfactory marker protein
OPRM1	opioid receptor, mu 1
ORAI1	ORAI calcium release-activated calcium modulator 1
OSMR	oncostatin M receptor
OXR1	oxidation resistance 1
P2RX7	purinergic receptor P2X, ligand gated ion channel, 7
P4HA1	prolyl 4-hydroxylase, alpha polypeptide I
PADI1	peptidyl arginine deiminase, type I
PAX3	paired box 3
PCDH7	protocadherin 7
PCLO	piccolo presynaptic cytomatrix protein
PCOLCE	procollagen C-endopeptidase enhancer
PCSK1	proprotein convertase subtilisin/kexin type 1
PCSK2	proprotein convertase subtilisin/kexin type 2
PCTP	phosphatidylcholine transfer protein
PDE3B	phosphodiesterase 3B, cGMP-inhibited
PDLIM2	PDZ and LIM domain 2 (mystique)
PENK	proenkephalin
PGR	progesterone receptor

PIM2	Pim-2 proto-oncogene, serine/threonine kinase
PIP5K1A	phosphatidylinositol-4-phosphate 5-kinase, type I, alpha
PLAC8	placenta-specific 8
PLAU	plasminogen activator, urokinase
PLAUR	plasminogen activator, urokinase receptor
PLCB1	phospholipase C, beta 1 (phosphoinositide-specific)
PLD1	phospholipase D1, phosphatidylcholine-specific
PLIN2	perilipin 2
PLIN3	perilipin 3
PLIN4	perilipin 4
PLIN5	perilipin 5
PLTP	phospholipid transfer protein
PLXNA2	plexin A2
PNPLA2	patatin-like phospholipase domain containing 2
POLR2C	polymerase (RNA) II (DNA directed) polypeptide C, 33kDa
PPARA	peroxisome proliferator-activated receptor alpha
PPIC	peptidylprolyl isomerase C (cyclophilin C)
PPP1R14A	protein phosphatase 1, regulatory (inhibitor) subunit 14A
PPP1R1B	protein phosphatase 1, regulatory (inhibitor) subunit 1B
PRC1	protein regulator of cytokinesis 1
PRDM16	PR domain containing 16
PRDX6	peroxiredoxin 6
Prh1/Prp2	proline rich protein HaeIII subfamily 1
PRKCD	protein kinase C, delta
PRKG2	protein kinase, cGMP-dependent, type II
PRL	prolactin
Prl2c2 (includes others)	prolactin family 2, subfamily c, member 2
PRLR	prolactin receptor
PROCR	protein C receptor, endothelial
PSIP1	PC4 and SFRS1 interacting protein 1
PSMB8	proteasome (prosome, macropain) subunit, beta type, 8
PSMB9	proteasome (prosome, macropain) subunit, beta type, 9
PTCRA	pre T-cell antigen receptor alpha
PTGS1	prostaglandin-endoperoxide synthase 1 (prostaglandin G/H synthase and cyclooxygenase)
PTGS2	prostaglandin-endoperoxide synthase 2 (prostaglandin G/H synthase and cyclooxygenase)
PTHLH	parathyroid hormone-like hormone
PTN	pleiotrophin
PTPN6	protein tyrosine phosphatase, non-receptor type 6
PTPRC	protein tyrosine phosphatase, receptor type, C
PTTG1	pituitary tumor-transforming 1
RAB5A	RAB5A, member RAS oncogene family
RAB7B	RAB7B, member RAS oncogene family
RAC2	ras-related C3 botulinum toxin substrate 2 (rho family, small GTP binding protein Rac2)

RARA	retinoic acid receptor, alpha
RARRES2	retinoic acid receptor responder (tazarotene induced) 2
RASA1	RAS p21 protein activator (GTPase activating protein) 1
RASGRF1	Ras protein-specific guanine nucleotide-releasing factor 1
RB1	retinoblastoma 1
RBP1	retinol binding protein 1, cellular
RDH5	retinol dehydrogenase 5 (11-cis/9-cis)
RECK	reversion-inducing-cysteine-rich protein with kazal motifs
REST	RE1-silencing transcription factor
RET	ret proto-oncogene
RGS16	regulator of G-protein signaling 16
RGS4	regulator of G-protein signaling 4
RHOB	ras homolog family member B
RIPK1	receptor (TNFRSF)-interacting serine-threonine kinase 1
RNF19B	ring finger protein 19B
RNF213	ring finger protein 213
RNF38	ring finger protein 38
RORA	RAR-related orphan receptor A
RPE65	retinal pigment epithelium-specific protein 65kDa
RRM1	ribonucleotide reductase M1
RSPO3	R-spondin 3
RTN4	reticulon 4
RTP4	receptor (chemosensory) transporter protein 4
RUNX1	runt-related transcription factor 1
RXRG	retinoid X receptor, gamma
S100A4	S100 calcium binding protein A4
S100A8	S100 calcium binding protein A8
S100A9	S100 calcium binding protein A9
SALL4	spalt-like transcription factor 4
SAR1A	secretion associated, Ras related GTPase 1A
SAT1	spermidine/spermine N1-acetyltransferase 1
Scd2	stearoyl-Coenzyme A desaturase 2
SCG2	secretogranin II
SCTR	secretin receptor
SDC4	syndecan 4
SDK1	sidekick cell adhesion molecule 1
SEC23A	Sec23 homolog A (S. cerevisiae)
SELE	selectin E
SELPLG	selectin P ligand
SEMA3C	sema domain, immunoglobulin domain (Ig), short basic domain, secreted, (semaphorin) 3C
SEMA3E	sema domain, immunoglobulin domain (Ig), short basic domain, secreted, (semaphorin) 3E
SERINC1	serine incorporator 1
SERINC2	serine incorporator 2
SERP1	stress-associated endoplasmic reticulum protein 1

SERPINA1	serpin peptidase inhibitor, clade A (alpha-1 antiproteinase, antitrypsin), member 1
SERPINA3	serpin peptidase inhibitor, clade A (alpha-1 antiproteinase, antitrypsin), member 3
Serpina3g (includes others)	serine (or cysteine) peptidase inhibitor, clade A, member 3G
SERPINE1	serpin peptidase inhibitor, clade E (nexin, plasminogen activator inhibitor type 1), member 1
SERPING1	serpin peptidase inhibitor, clade G (C1 inhibitor), member 1
SETD2	SET domain containing 2
SF3B3	splicing factor 3b, subunit 3, 130kDa
SFI1	Sfi1 homolog, spindle assembly associated (yeast)
SGK1	serum/glucocorticoid regulated kinase 1
SGSM3	small G protein signaling modulator 3
SH3RF1	SH3 domain containing ring finger 1
SHH	sonic hedgehog
SIRT1	sirtuin 1
SKIL	SKI-like proto-oncogene
SLC11A1	solute carrier family 11 (proton-coupled divalent metal ion transporter), member 1
SLC18A3	solute carrier family 18 (vesicular acetylcholine transporter), member 3
SLC22A4	solute carrier family 22 (organic cation/zwitterion transporter), member 4
SLC27A5	solute carrier family 27 (fatty acid transporter), member 5
SLC29A1	solute carrier family 29 (equilibrative nucleoside transporter), member 1
SLC2A3	solute carrier family 2 (facilitated glucose transporter), member 3
SLC2A4	solute carrier family 2 (facilitated glucose transporter), member 4
SLC2A5	solute carrier family 2 (facilitated glucose/fructose transporter), member 5
SLC31A1	solute carrier family 31 (copper transporter), member 1
SLC38A2	solute carrier family 38, member 2
SLC39A10	solute carrier family 39 (zinc transporter), member 10
SLC3A1	solute carrier family 3 (amino acid transporter heavy chain), member 1
SLC4A7	solute carrier family 4, sodium bicarbonate cotransporter, member 7
SLC6A12	solute carrier family 6 (neurotransmitter transporter), member 12
SLC6A6	solute carrier family 6 (neurotransmitter transporter), member 6
SLC7A11	solute carrier family 7 (anionic amino acid transporter light chain, xc- system), member 11
SLC7A5	solute carrier family 7 (amino acid transporter light chain, L system), member 5
SLC9A3	solute carrier family 9, subfamily A (NHE3, cation proton antiporter 3), member 3
SLFN5	schlafen family member 5
SLPI	secretory leukocyte peptidase inhibitor
SMAD9	SMAD family member 9
SMAGP	small cell adhesion glycoprotein
SMARCC1	SWI/SNF related, matrix associated, actin dependent regulator of chromatin, subfamily c, member 1
SMC2	structural maintenance of chromosomes 2

SMO	smoothened, frizzled class receptor
SNRPN	small nuclear ribonucleoprotein polypeptide N
SOCS3	suppressor of cytokine signaling 3
SOD3	superoxide dismutase 3, extracellular
SORCS3	sortilin-related VPS10 domain containing receptor 3
SOX9	SRY (sex determining region Y)-box 9
SP110	SP110 nuclear body protein
SPAG4	sperm associated antigen 4
SPARC	secreted protein, acidic, cysteine-rich (osteonectin)
SPOCK1	sparc/osteonectin, cwcv and kazal-like domains proteoglycan (testican) 1
SPOP	speckle-type POZ protein
SPP1	secreted phosphoprotein 1
SQRDL	sulfide quinone reductase-like (yeast)
SRC	SRC proto-oncogene, non-receptor tyrosine kinase
SREBF1	sterol regulatory element binding transcription factor 1
SREBF2	sterol regulatory element binding transcription factor 2
SRP54	signal recognition particle 54kDa
SRSF3	serine/arginine-rich splicing factor 3
SSBP1	single-stranded DNA binding protein 1, mitochondrial
SST	somatostatin
SSTR2	somatostatin receptor 2
ST18	suppression of tumorigenicity 18, zinc finger
ST3GAL1	ST3 beta-galactoside alpha-2,3-sialyltransferase 1
STAT3	signal transducer and activator of transcription 3 (acute-phase response factor)
STK10	serine/threonine kinase 10
STMN1	stathmin 1
STRA6	stimulated by retinoic acid 6
STX3	syntaxin 3
STXBP5	syntaxin binding protein 5 (tomosyn)
SYCP1	synaptonemal complex protein 1
SYNPO	synaptopodin
TAP1	transporter 1, ATP-binding cassette, sub-family B (MDR/TAP)
TAP2	transporter 2, ATP-binding cassette, sub-family B (MDR/TAP)
TAPBP	TAP binding protein (tapasin)
TBP	TATA box binding protein
TBX21	T-box 21
TBXAS1	thromboxane A synthase 1 (platelet)
TCF12	transcription factor 12
TCF3	transcription factor 3
TDGF1	teratocarcinoma-derived growth factor 1
TEAD1	TEA domain family member 1 (SV40 transcriptional enhancer factor)
TERT	telomerase reverse transcriptase
TF	transferrin
TFRC	transferrin receptor
TGFB1	transforming growth factor, beta 1

TGFB2	transforming growth factor, beta 2
TGFBI	transforming growth factor, beta-induced, 68kDa
TGFBR1	transforming growth factor, beta receptor 1
TGFBR2	transforming growth factor, beta receptor II (70/80kDa)
TGM1	transglutaminase 1
TGM2	transglutaminase 2
Tgtp1/Tgtp2	T cell specific GTPase 1
TH	tyrosine hydroxylase
THBD	thrombomodulin
THOC1	THO complex 1
THY1	Thy-1 cell surface antigen
TIMP1	TIMP metallopeptidase inhibitor 1
TIMP2	TIMP metallopeptidase inhibitor 2
TK1	thymidine kinase 1, soluble
TLR2	toll-like receptor 2
TLR4	toll-like receptor 4
TLR6	toll-like receptor 6
TM4SF1	transmembrane 4 L six family member 1
TMEM176A	transmembrane protein 176A
TMEM176B	transmembrane protein 176B
TMEM98	transmembrane protein 98
Tmsb4x (includes others)	thymosin, beta 4, X chromosome
TNFAIP6	tumor necrosis factor, alpha-induced protein 6
TNFAIP8	tumor necrosis factor, alpha-induced protein 8
TNFRSF1A	tumor necrosis factor receptor superfamily, member 1A
TNNC2	troponin C type 2 (fast)
TP63	tumor protein p63
TP73	tumor protein p73
TPM3	tropomyosin 3
TRAF2	TNF receptor-associated factor 2
TRAF6	TNF receptor-associated factor 6, E3 ubiquitin protein ligase
TREM1	triggering receptor expressed on myeloid cells 1
TRH	thyrotropin-releasing hormone
TRIM14	tripartite motif containing 14
TRPC6	transient receptor potential cation channel, subfamily C, member 6
TRPM3	transient receptor potential cation channel, subfamily M, member 3
TSPO	translocator protein (18kDa)
TTF2	transcription termination factor, RNA polymerase II
TWIST1	twist family bHLH transcription factor 1
TYMP	thymidine phosphorylase
UBA7	ubiquitin-like modifier activating enzyme 7
Ubb	ubiquitin B
UBD	ubiquitin D
UBL3	ubiquitin-like 3
UCP2	uncoupling protein 2 (mitochondrial, proton carrier)
UCP3	uncoupling protein 3 (mitochondrial, proton carrier)

UFD1L	ubiquitin fusion degradation 1 like (yeast)
UGT1A6	UDP glucuronosyltransferase 1 family, polypeptide A6
USP18	ubiquitin specific peptidase 18
UTRN	utrophin
VAMP5	vesicle-associated membrane protein 5
VAMP8	vesicle-associated membrane protein 8
VAV3	vav 3 guanine nucleotide exchange factor
VCAM1	vascular cell adhesion molecule 1
VEGFA	vascular endothelial growth factor A
VEGFC	vascular endothelial growth factor C
VIM	vimentin
VIP	vasoactive intestinal peptide
VLDLR	very low density lipoprotein receptor
VPREB1	pre-B lymphocyte 1
VSNL1	visinin-like 1
VTI1A	vesicle transport through interaction with t-SNAREs 1A
Wap	whey acidic protein
WARS	tryptophanyl-tRNA synthetase
WEE1	WEE1 G2 checkpoint kinase
WHSC1	Wolf-Hirschhorn syndrome candidate 1
WISP1	WNT1 inducible signaling pathway protein 1
WNT10A	wingless-type MMTV integration site family, member 10A
WNT9A	wingless-type MMTV integration site family, member 9A
WTAP	Wilms tumor 1 associated protein
XAF1	XIAP associated factor 1
XDH	xanthine dehydrogenase
XIAP	X-linked inhibitor of apoptosis, E3 ubiquitin protein ligase
YWHAE	tyrosine 3-monooxygenase/tryptophan 5-monooxygenase activation protein, epsilon
ZFP36L1	ZFP36 ring finger protein-like 1
ZNF367	zinc finger protein 367

Dysregulated transcripts in the adult brain of 9 month-old 5XFAD transgenic female mice i[illegible] comparison with wild type mice. The acronym and the full name of each gene are indicated. Genes wit[illegible] a VDRE are highlighted in grey.
