# Supplementary figures and images for: Vitamin D interacts with Esr1 and Igf1 to regulate molecular pathways relevant to Alzheimer’s disease
Source: Mol Neurodegener. 2016 Mar 1;11:22. doi: 10.1186/s13024-016-0087-2 (PMC4774101; doi:10.1186/s13024-016-0087-2)

Wild type

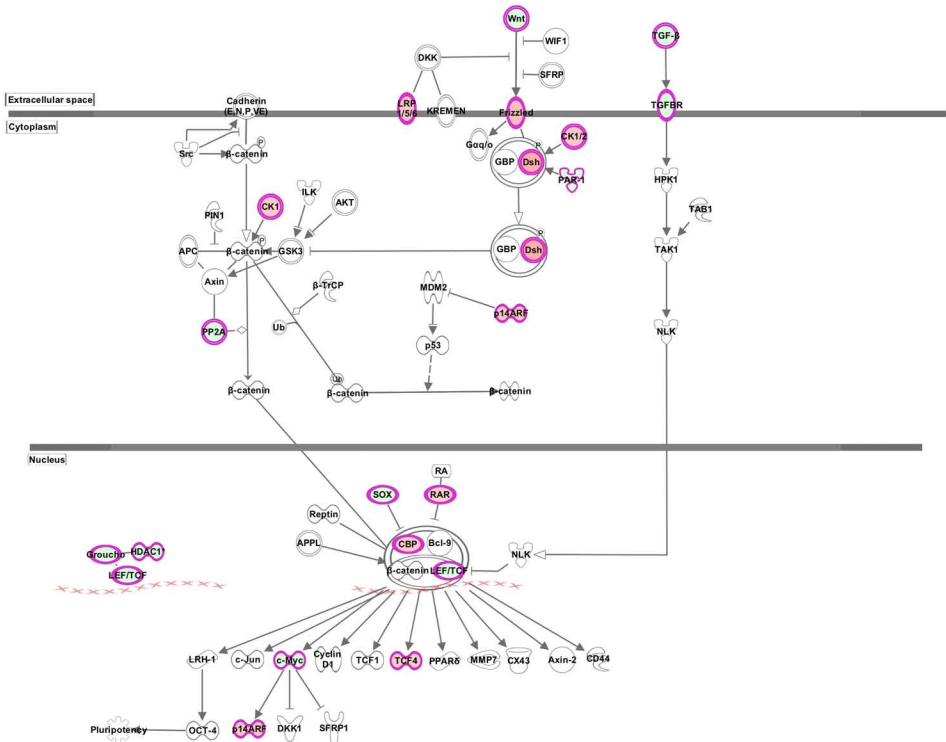

Transgenic

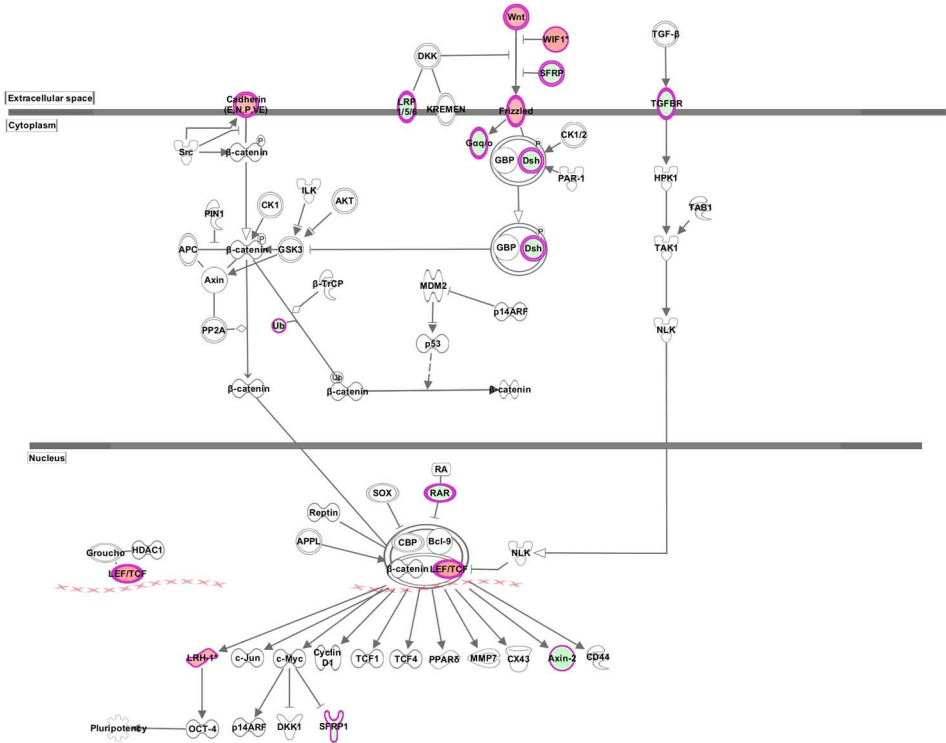

Supplement: Additional file 6: — Figure S1. Comparison of the different DEGs affected in the Wnt/β-catenin signaling pathway according to mouse genotype (Wt VitD versus Tg VitD). (PDF 219 kb) [file 13024_2016_87_MOESM6_ESM.pdf]
